# Sleep Deprivation Induces Glial Dysfunction, Synaptic Loss, Metabolic Imbalance, and Cognitive Impairment in 12‐Month‐Old Mice: Protective Effects of D30

**DOI:** 10.1002/cns.71134

**Published:** 2026-09-10

**Authors:** Xueyan Liu, Huiling Lin, Jian Zhong, Xili Chen, Zonglin Wang, Meng Shi, Hang Shi, Hui Zhang, Ping Chen, Zu‐Cheng Ye

**Affiliations:** ^1^ School of Pharmacy, Fujian Medical University Fuzhou China; ^2^ School of Basic Medical Sciences, Fujian Medical University Fuzhou China; ^3^ Fujian Medical University Fuzhou China; ^4^ Department of Anesthesiology First Affiliated Hospital of Fujian Medical University Fuzhou China

**Keywords:** cognitive impairment, glial dysfunction, metabolic dysregulation, sleep deprivation, synaptic dysfunction

## Abstract

**Background:**

Sleep deprivation (SD) has been increasingly implicated in age‐related cognitive decline. However, the mechanisms linking SD duration to progressive disruption of glial homeostasis, synaptic vulnerability, and metabolic dysregulation remain poorly defined.

**Methods:**

We employed 12‐month‐old Thy1‐EGFP and Cx3CR1‐EGFP mice to investigate the effects of short‐term sleep deprivation (SSD) and long‐term sleep deprivation (LSD) on neuronal architecture, microglial morphology, and astrocytic homeostasis. Quantitative analyses included dendritic spine density, synaptic protein expression, glial morphological and transcriptional markers, and untargeted plasma metabolomics. The neuroprotective effects of D30, a novel small molecule compound, were also evaluated.

**Results:**

SSD induced relatively transient oxidative stress and glial suppression, whereas LSD led to sustained reductions in dendritic spine density, synaptic protein levels, and microglial/astrocytic morphological complexity. LSD further disrupted mitochondrial metabolism, notably involving the TCA cycle, AMPK‐associated signaling, and lipid homeostasis. Treatment with D30 significantly ameliorated LSD‐induced deficits by preserving glial homeostatic features, restoring synaptic protein expression, maintaining dendritic spine density, and rebalancing systemic metabolism, ultimately improving cognitive performance.

**Conclusions:**

LSD impairs glial homeostatic integrity and synaptic stability in association with systemic metabolic dysfunction in middle‐aged mice. D30 effectively alleviates these impairments, highlighting its potential as a protective intervention for chronic SD‐related neurocognitive dysfunction.

## Graphical abstract

Sleep deprivation induces neuroglial atrophy and dendritic spine loss, accompanied by metabolic disturbances involving 1‐methyladenosine, citric acid, L‐kynurenine, and berberine, indicating disrupted neuroenergetic and immunometabolic homeostasis. These alterations result in deficits in spatial recognition, fear conditioning, nesting, and Morris Water Maze performance. D30 markedly alleviates these changes by preserving synaptic and glial integrity, restoring metabolic balance, and maintaining cognitive function.

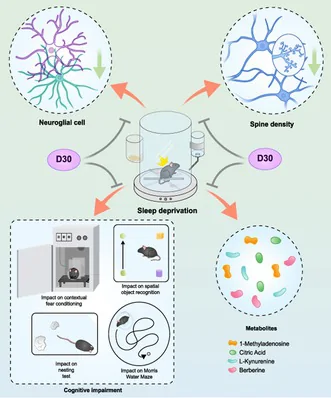

## 1 Introduction

Sleep is a fundamental biological process essential for cognitive function, metabolic regulation, and neuroprotection. Chronic disruption of sleep architecture, particularly in aging populations, has been recognized as a major risk factor for cognitive decline and neurodegeneration‐related disorders [1, 2]. Sleep deprivation (SD) impairs multiple homeostatic processes in the brain, including synaptic remodeling, glial homeostasis, and metabolic waste clearance. However, the precise mechanisms by which SD contributes to neurocognitive impairments—particularly its effects on synaptic plasticity, glial homeostasis, and metabolic regulation—remain incompletely understood.

Growing evidence highlights the intricate interplay between sleep and brain health. Sleep is vital for memory consolidation, regulation of neuroinflammation, and clearance of neurotoxic waste via the glymphatic system. Disruption of these processes can trigger synaptic instability, oxidative stress, and metabolic imbalance, all of which are key contributors to aging‐related cognitive decline [3, 4, 5]. While SD has been widely investigated in transgenic models of Alzheimer's disease (e.g., APP/PS1 mice), where it exacerbates amyloid‐β (Aβ) pathology, recent studies have shown that chronic sleep fragmentation also impairs cognition and elevates neuroinflammatory markers in wild‐type mice, even in the absence of genetic susceptibility [6, 7, 8, 9].

The mechanisms underlying SD‐induced cognitive decline are multifactorial, involving synaptic dysfunction, metabolic derangements, and glial abnormalities [10, 11, 12, 13]. Glial cells—particularly astrocytes and microglia—play indispensable roles in maintaining neuronal health. Microglia are key regulators of neuroinflammation and immune surveillance, while astrocytes support synaptic function, metabolic balance, and clearance of brain waste [14, 15, 16]. SD perturbs these glial functions by impairing microglial surveillance and diminishing astrocytic support [17, 18]. Moreover, SD disrupts neuronal energy homeostasis, which is critical for synaptic plasticity and higher‐order cognitive functions such as learning and memory [19, 20]. In rodent models, SD reduces dendritic spine density, shortens dendritic length, and decreases prefrontal cortex volume—changes that strongly correlate with memory deficits and glial dysfunction [21, 22]. These findings indicate that SD can affect neuronal structure and glial function in a brain region‐ and duration‐dependent manner. Such glial disturbances may initiate neuropathological cascades that contribute to neurodegeneration‐related synaptic vulnerability and progressive synaptic loss [23, 24].

Importantly, the effects of SD are age dependent. Prior studies have shown that SD induces divergent behavioral and physiological responses in young versus aged mice [25]. Specifically, SD increases microglial density and morphological complexity in young mice but reduces these features in aged mice, suggesting an age‐related vulnerability of glial cells to sleep loss [25]. In addition, SD activates the DNA damage response and senescence‐associated secretory phenotype—hallmarks of cellular senescence that accelerate aging processes [26]. SD also exacerbates metabolic dysregulation in younger adults, inducing premature aging‐like phenotypes [27]. These findings underscore the need for targeted interventions in middle‐aged populations, who may be particularly susceptible to SD‐induced brain dysfunction.

To investigate the cellular impact of SD, we employed middle‐aged (12‐month‐old) Thy1‐EGFP and Cx3CR1‐EGFP transgenic mice, which enable high‐resolution visualization of neurons and microglia, respectively. The Thy1‐EGFP model expresses enhanced green fluorescent protein (EGFP) in projection neurons, allowing for precise assessment of dendritic architecture and synaptic integrity [28, 29, 30]. In contrast, the Cx3CR1‐EGFP model selectively labels microglia, facilitating detailed analyses of microglial morphology and surveillance capacity in response to SD [31, 32].

Although SD can affect multiple brain regions, including the cortex, hypothalamus, amygdala, striatum, and hippocampus, the present study focused primarily on the hippocampus because of its central role in learning, memory, emotional regulation, synaptic plasticity, and adult neurogenesis.

**FIGURE 1:**
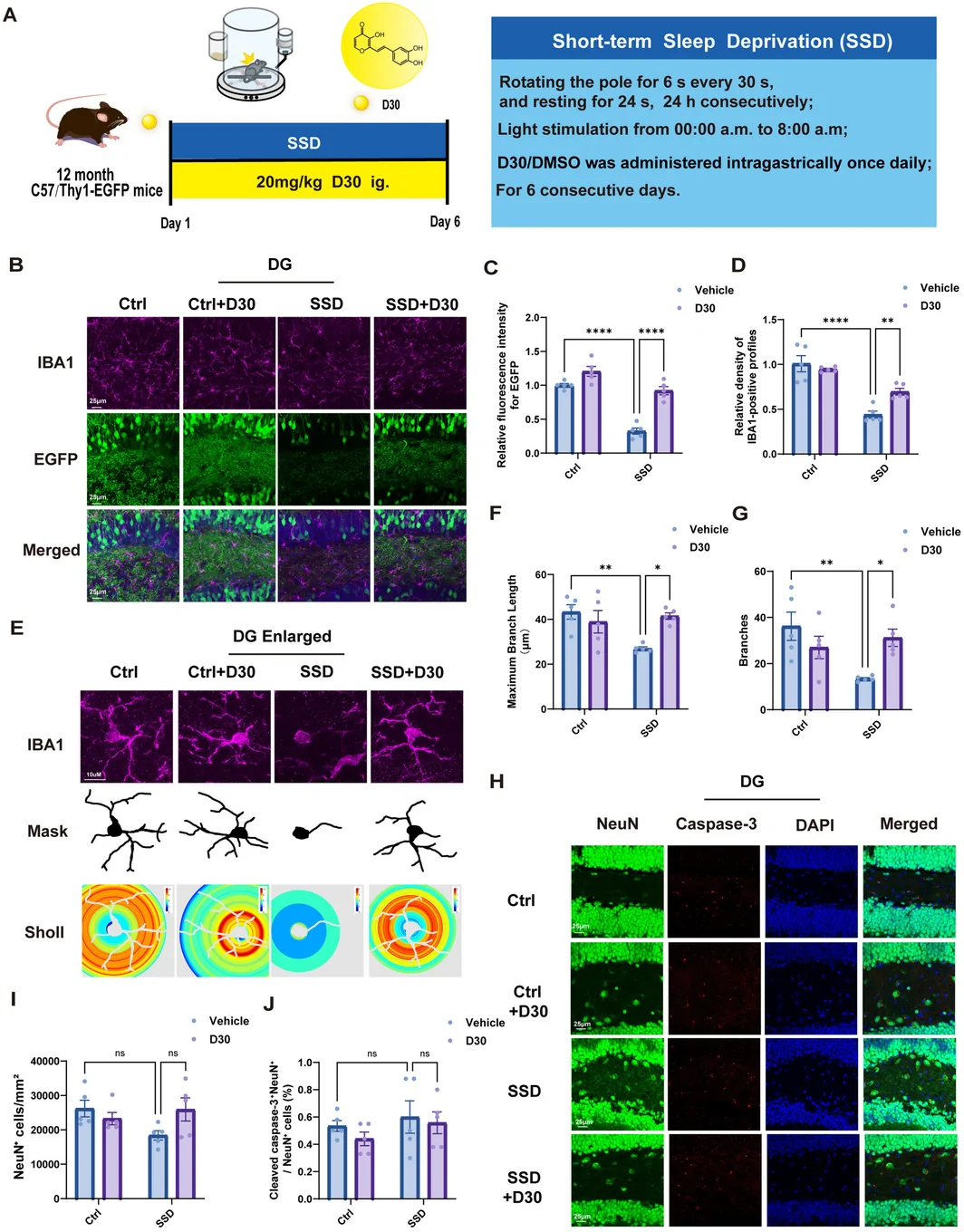
D30 attenuates SSD‐induced reductions in Thy1‐EGFP fluorescence and microglial morphological complexity without overt neuronal loss or apoptosis in the dentate gyrus of middle‐aged mice. (A) Schematic illustration of the short‐term sleep deprivation (SSD) protocol and D30 administration. Twelve‐month‐old C57BL/6 and Thy1‐EGFP mice were subjected to SSD for 6 consecutive days using a rotating platform paradigm, and D30 or vehicle was administered intragastrically once daily at 20 mg/kg. (B) Representative immunofluorescence images of IBA1 and Thy1‐EGFP in the dentate gyrus (DG) from Ctrl, Ctrl + D30, SSD, and SSD + D30 Thy1‐EGFP mice. SSD reduced IBA1 immunoreactivity and Thy1‐EGFP fluorescence, whereas D30 treatment partially attenuated these changes. Scale bars, 25 μm. (C, D) Quantification of relative Thy1‐EGFP fluorescence intensity (C) and the relative density of IBA1‐positive profiles (D) in the DG of Thy1‐EGFP mice. (E) Representative high‐magnification images of IBA1‐positive microglia, corresponding binary masks, and Sholl analysis heatmaps from Thy1‐EGFP mice. Scale bars, 10 μm. (F, G) Quantification of maximum branch length (F) and number of microglial branches (G) based on morphological analysis in Thy1‐EGFP mice. SSD markedly reduced microglial process complexity, whereas D30 administration partially restored microglial branching complexity. (H) Representative immunofluorescence images of NeuN, cleaved caspase‐3, and DAPI in the DG from Ctrl, Ctrl + D30, SSD, and SSD + D30 C57BL/6 mice. Scale bars, 25 μm. (I, J) Quantification of NeuN‐positive cell density (I) and the percentage of cleaved caspase‐3‐positive NeuN‐positive cells among total NeuN‐positive cells (J) in C57BL/6 mice. No significant neuronal loss or increase in neuronal apoptosis was detected following SSD, indicating that the SSD‐induced reduction in Thy1‐EGFP fluorescence was not attributable to overt neuronal loss or caspase‐3‐mediated apoptosis under the present experimental conditions. Data are presented as mean ± SEM (*n* = 5 animals per group). Statistical significance was assessed using ordinary two‐way ANOVA with experimental condition (Ctrl vs. SSD) and treatment (vehicle vs. D30) as factors, followed by Šídák's multiple‐comparisons test. For (C), significant main effects of experimental condition (*F*(1,16) = 79.45, *p* < 0.0001) and treatment (*F*(1,16) = 55.36, *p* < 0.0001), as well as a significant experimental condition × treatment interaction (*F*(1,16) = 13.18, *p* = 0.0022), were detected. For (D), the main effect of experimental condition was significant (*F*(1,16) = 59.84, *p* < 0.0001), whereas the main effect of treatment was not (*F*(1,16) = 3.310, *p* = 0.0876); the interaction was significant (*F*(1,16) = 9.169, *p* = 0.0080). For (F), the main effect of experimental condition was significant (*F*(1,16) = 5.000, *p* = 0.0399), whereas the main effect of treatment was not (*F*(1,16) = 2.701, *p* = 0.1198); the interaction was significant (*F*(1,16) = 9.343, *p* = 0.0075). For (G), the main effect of experimental condition was significant (*F*(1,16) = 4.556, *p* = 0.0486), whereas the main effect of treatment was not (*F*(1,16) = 0.9739, *p* = 0.3384); the interaction was significant (*F*(1,16) = 9.600, *p* = 0.0069). For (I), neither the main effect of experimental condition (*F*(1,16) = 1.202, *p* = 0.2892) nor that of treatment (*F*(1,16) = 0.9525, *p* = 0.3436) was significant, whereas the interaction was significant (*F*(1,16) = 4.962, *p* = 0.0406). For (J), neither the main effect of experimental condition (*F*(1,16) = 1.380, *p* = 0.2574), the main effect of treatment (*F*(1,16) = 0.7537, *p* = 0.3981), nor their interaction (*F*(1,16) = 0.1102, *p* = 0.7442) was significant. ns, not significant; *p < 0.05, **p < 0.01, and ****p < 0.0001 for the indicated post hoc comparisons.

Additionally, we evaluated the neuroprotective potential of D30 (chemical structure shown in Figure 1A), a small‐molecule compound reported to modulate glial homeostasis and cognitive function. D30 has been shown to cross the blood–brain barrier and exhibits effects on glial homeostasis [33, 34, 35]; however, its ability to attenuate SD‐induced neuropathology remains unexplored. This study aims to elucidate the molecular and cellular mechanisms underlying SD‐induced brain dysfunction in middle‐aged mice and to assess the protective efficacy of D30 by examining the interplay among glial dysregulation, synaptic deterioration, and systemic metabolic disruption.

## 2 Material and Methods

### 2.1 Animals

Thy1‐EGFP transgenic mice (strain #007788, RRID:IMSR_JAX:007788) and Cx3CR1‐EGFP transgenic mice (strain #005582, RRID:IMSR_JAX:005582) were obtained from The Jackson Laboratory (Bar Harbor, ME, USA) and bred in our Specific Pathogen‐Free (SPF) facility. Age‐matched wild‐type (WT) C57BL/6 mice were purchased from Yao Kang Biological Technology (Guangzhou, China). All animals were 12 months old (28–32 g).

Only male mice were used to minimize the differences caused by fluctuations in the estrus cycle [36]. Such variability could confound treatment effects under sleep‐deprivation paradigms; therefore, males were selected to ensure consistency and reduce biological noise. Mice were housed 6 per cage under controlled temperature, humidity, and a 12 h light/dark cycle, with ad libitum food and water. All procedures were approved by the Institutional Animal Care and Use Committee of Fujian Medical University (IACUC FJMU 2024‐0274).

### 2.2 Animal Models and Experimental Design

In this study, short‐term sleep deprivation (SSD) was defined as a 6‐day period of continuous sleep restriction, whereas long‐term sleep deprivation (LSD) referred to a 28‐day prolonged deprivation protocol. Unless otherwise specified, SD in this manuscript refers to both paradigms.

#### 2.2.1 SSD Thy1‐EGFP Mouse Model

To evaluate the effects of SSD and the neuroprotective potential of compound D30, we employed the Thy1‐EGFP mouse model. Mice were randomly assigned to four experimental groups (*n* = 6 per group): Control + DMSO (Ctrl + DMSO); Control + D30 (Ctrl + D30); SSD + DMSO (SSD + DMSO); SSD + D30 (SSD + D30). Compound D30 was initially dissolved in DMSO at a concentration of 40 mg/mL, then diluted with distilled water to a final concentration of 2 mg/mL. It was administered once daily via oral gavage at a dose of 20 mg/kg/day, a dosage selected based on our previous studies [33, 34, 35]. Control mice received vehicle solution containing 5% DMSO in distilled water. The administration volume was adjusted according to each animal's body weight, which was monitored every other day throughout the experiment. Mice were housed under standardized conditions (temperature: 23°C–25°C, humidity: 60%, and a 12‐h light/dark cycle).

The SSD protocol was based on relevant previous studies with appropriate modifications [37, 38, 39]. It consisted of continuous low‐speed 360° pole rotation lasting for 6 s every 30 s, followed by a 24 s pause, and was maintained throughout the day to disrupt sustained sleep episodes. In addition, a mild light stimulus was applied daily from 00:00 to 08:00 as an auxiliary environmental manipulation.

#### 2.2.2 SSD C57BL/6 Mouse Model

To assess neuronal integrity and changes in hippocampal gene expression following sleep deprivation, C57BL/6 mice were used. Mice were randomly divided into four experimental groups (*n* = 10 per group): Control + vehicle (Ctrl), Control + D30 (Ctrl + D30), SSD + vehicle (SSD), and SSD + D30. All animals were housed under standard SPF conditions (temperature: 23°C–25°C; humidity: 60%; light/dark cycle: 12 h/12 h), with ad libitum access to food and water. The SSD protocol and D30 treatment regimen were identical to those described for the Thy1‐EGFP mouse model.

#### 2.2.3 SSD Cx3CR1‐EGFP Mouse Model

To further investigate microglial alterations induced by sleep deprivation, Cx3CR1‐EGFP transgenic mice were used. Mice were randomly divided into two experimental groups (*n* = 6 per group): Control (Ctrl) and SSD. All animals were housed under standard SPF conditions (temperature: 23°C–25°C; humidity: 60%; light/dark cycle: 12 h/12 h), with ad libitum access to food and water. The SSD protocol applied to Cx3CR1‐EGFP mice was identical to that described for the Thy1‐EGFP model, involving continuous low‐speed pole rotation and nocturnal light stimulation.

#### 2.2.4 LSD Thy1‐EGFP Mouse Model

To assess the long‐term effects of sleep deprivation and the protective potential of D30 during chronic sleep deprivation, Thy1‐EGFP mice were subjected to a 28‐day LSD protocol followed by behavioral assessments. Mice were randomly assigned to four groups (*n* = 10 per group): Control + DMSO (Ctrl + DMSO), Control + D30 (Ctrl + D30), LSD + DMSO (LSD + DMSO), and LSD + D30 (LSD + D30). The D30 treatment regimen (20 mg/kg/day via oral gavage) was identical to that used in the SSD model and continued throughout the entire 28‐day experimental period.

The LSD protocol was designed as a prolonged and repeated sleep disruption paradigm. Mechanical sleep disturbance was applied for approximately 20 h per day, consisting of continuous low‐speed 360° pole rotation, while mice were allowed a defined daily rest period from 08:00 to 12:00, during which no mechanical or light‐based stimuli were administered. This rest window was included to minimize excessive physical fatigue while maintaining sustained sleep disruption across days.

In addition to mechanical stimulation, a mild light stimulus was applied during the dark phase (from 00:00 to 08:00) as an auxiliary environmental manipulation to interfere with sleep continuity and reinforce sleep fragmentation, rather than to serve as a primary arousing signal. This combined paradigm was maintained throughout the LSD period to induce cumulative sleep disruption.

Behavioral testing began after the first week of LSD. To minimize acute fatigue‐related confounding, mice were allowed an 18‐h recovery interval before each behavioral test, after which the LSD protocol was resumed. The tests were conducted in the following sequence: Elevated Plus Maze (EPM), Tail Suspension Test (TST), Nesting Test, Open Field (OF) test, Novel Object Recognition (NOR), Fear Conditioning Test (FCT), and Morris Water Maze (MWM). Behavioral assessments spanned the second and third weeks, with at least 20 h of SD between different tests to minimize cumulative behavioral interference. During the final week, mechanical sleep disruption was suspended during MWM training and testing periods to avoid direct interference with spatial learning assessment.

All mice received daily oral gavage of D30 or the control vehicle throughout the experiment, and body weights were monitored every other day. No mortality was observed throughout the experimental period.

### 2.3 Behavioral Experiments

Elevated Plus Maze (EPM). The maze had two open and two closed arms (60 × 5 cm; closed‐wall height 25 cm) elevated 50 cm, with a 5 × 5 cm center. After 30‐min acclimation, each mouse was tested for 5 min and tracked (Jiangsu SANS, Nanjing, China). Time in open arms and open‐arm entries were recorded. The apparatus was cleaned with 75% ethanol between trials.

Tail Suspension Test (TST). Mice were acclimated for 60 min. Each mouse was suspended by adhesive tape ~1 cm from the tail tip, ~40 cm above the floor, for 5 min in a chamber with soft backlighting and visual isolation. Immobility (%) was quantified in ANY‐maze software (version 7.51, Stoelting Co., Wood Dale, IL, USA) as an index of behavioral despair/depressive‐like behavior.

Nesting Test. After 1‐h acclimation to single housing, mice received one pressed cotton square (5 × 5 × 0.5 cm; Yuyan, Shanghai). After 24 h, nests were scored 1–5 and remaining unshredded material was weighed.

Open Field (OF). Mice were tested individually in a 50 × 50 × 50 cm arena (SANSbio, Jiangsu, China) for 5 min. Entries/time in the center and total distance were analyzed in ANY‐maze. The arena was cleaned with 75% ethanol between trials.

Novel Object Recognition (NOR). Day 1: after 5 min of free exploration, mice were exposed for 5 min to two identical objects. Day 2: one object was replaced with a novel object at the same locations; exploration was recorded for 5 min. Discrimination index: *R*% = *T*new/(*T*new + *T*familiar) × 100.

Fear Conditioning Test (FCT). Training (Day 1): after 2‐min acclimation, a 30‐s 80 dB tone was paired with a 2‐s 0.7 mA foot shock, repeated twice (2‐min inter‐trial interval). Context test (Day 2): 5‐min exposure to the original chamber (no tone/shock). Cue test (Day 3): 3‐min exposure to a novel context followed by a 3‐min tone. Freezing behavior was automatically quantified using ANY‐maze software. For cue testing, context was altered using foam boards and 100 mL of 1%–1.5% acetic acid.

Morris Water Maze (MWM). A 120‐cm pool (water 22°C ± 1°C) with a hidden platform (7.5‐cm diameter) submerged 1 cm in quadrant IV and distal visual cues on the walls was used. After a 60‐s visible platform pretraining swim, mice underwent 6 days of acquisition from varied start points (60‐s trial; guided to the platform if not found; 15‐s stay). For the probe trial, the platform was removed; platform crossings and escape latency were analyzed in ANY‐maze.

### 2.4 Brain Tissue Collection

Mice were deeply anesthetized with avertin/2,2,2‐tribromoethanol (350 mg/kg, i.p.), and blood samples were collected via retro‐orbital sinus puncture. After blood collection, mice assigned for biochemical and metabolomic analyses were transcardially perfused with ice‐cold phosphate‐buffered saline (PBS), and brain tissues were rapidly dissected and snap‐frozen in liquid nitrogen. Mice assigned for immunofluorescence analysis were perfused with PBS, followed by 4% paraformaldehyde (PFA), and post‐fixed in 4% PFA overnight at 4°C. After perfusion, brains were processed for cryoprotection in a sucrose gradient and subsequently stored at −80°C until sectioning.

### 2.5 Sectioning and Sample Selection

Serial coronal sections were collected through the hippocampal region using a vibratome at a thickness of 40 μm. Sections containing the dorsal hippocampus were identified according to consistent anatomical landmarks. To ensure comparable sampling across animals and experimental groups, hippocampal sections were selected using a systematic 1‐in‐6 interval sampling strategy, corresponding to an interval of approximately 240 μm between analyzed sections. Adjacent sections were not used for the same quantitative endpoint.

For each animal, four anatomically matched, non‐adjacent dorsal hippocampal sections containing clearly defined CA1 and DG subregions were selected for staining and quantitative analysis, and the averaged value from these sections was used as one biological replicate.

Sections were included only when the hippocampal structure was intact and the CA1 and DG regions were clearly identifiable. Sections with tissue tearing, folding, incomplete hippocampal anatomy, uneven staining, severe background signal, or obvious imaging artifacts were excluded from analysis. All groups were processed in parallel using the same staining and imaging conditions. Section selection, image acquisition, and quantitative analyses were performed using identical criteria across groups, and investigators were blinded to group allocation during image analysis.

### 2.6 Immunofluorescence Analysis

To block non‐specific binding, sections were incubated with PBS containing 0.1% Triton X‐100 (PBST) and 5% normal goat serum. The following primary antibodies were used: anti‐IBA1 (rabbit, Wako, Osaka, Japan, Cat# 019‐19741, RRID:AB_839504, 1:1000), anti‐GFAP (rabbit, Abcam, Cambridge, UK, Cat# ab7260, RRID:AB_305808, 1:1500), anti‐PSD95 (rabbit, Cell Signaling Technology, Danvers, MA, USA, Cat# 3450S, RRID:AB_2292883, 1:100), anti‐NeuN (mouse, Abcam, Cambridge, UK, Cat# ab104224, RRID:AB_10711153, 1:1000), and cleaved caspase‐3 (Asp175) (5A1E) rabbit monoclonal antibody (Cell Signaling Technology, Danvers, MA, USA, Cat# 9664, RRID:AB_2070042, 1:500). Primary antibodies were applied overnight at 4°C. After three 10‐min washes with PBST, sections were incubated with the appropriate fluorophore‐conjugated secondary antibodies for 2 h at room temperature, including Goat anti‐Rabbit 647 (1:500, Jackson, Cat# 111‐605‐144, RRID:AB_2338078), Goat anti‐Rabbit 554 (1:500, Abcam, Cat# ab150086, RRID:AB_2890032), and Goat Anti‐Mouse 488 (1:500, Jackson Cat# 115‐546‐068, RRID:AB_2338864). Sections were then washed three times with PBST and mounted on glass slides with antifade mounting medium.

#### 2.6.1 Imaging of Microglia and Astrocytes

Confocal imaging of microglia and astrocytes was performed using a TCS SP8 confocal laser scanning microscope (Leica Microsystems, Germany) equipped with a 63× oil immersion objective. Hippocampal CA1 and DG regions were first identified using low‐magnification overview scans acquired at 0.75× digital zoom. High‐resolution imaging was subsequently performed with progressive digital zoom up to 4×.

Z‐stack images were acquired with 21 optical sections at 1 μm step size (total depth: 20 μm). The pinhole was set to 0.36 Airy units to optimize optical sectioning, and images were collected at a resolution of 1024 × 1024 pixels with a line averaging of 2. All imaging parameters were kept constant across experimental groups.

For morphological analysis, five mice per group were randomly selected. All image acquisition and subsequent analyses were performed under blinded conditions.

Image analysis was performed using Fiji/ImageJ (v1.53). IBA1‐ and GFAP‐stained images were converted to 8‐bit format prior to analysis. When necessary, images were denoised using the Despeckle function and contrast was enhanced using the Unsharp Mask filter. Microglial and astrocytic structures were segmented using consistent thresholding (Image → Adjust → Threshold) and converted into binary masks. Individual microglia were selected based on predefined criteria to ensure isolation of non‐overlapping cells.

Microglial morphology was quantified using the Skeletonize (2D/3D) plugin followed by the Analyze Skeleton (2D/3D) plugin, allowing measurement of total process length, branch number, branch points, and endpoints. Sholl analysis was performed using the Sholl Analysis plugin, with the soma defined as the center point and concentric circles generated at 5 μm intervals over a radial range of 5–100 μm to assess branching complexity.

GFAP fluorescence intensity and GFAP‐positive area fraction were quantified using identical threshold settings across all groups. Spatial calibration was standardized using the ImageJ calibration tool. For each animal, multiple non‐overlapping fields were analyzed and averaged as a single biological replicate.

#### 2.6.2 Imaging of CA1 Pyramidal Neurons

EGFP‐positive pyramidal neurons in the dorsal CA1 region of Thy1‐EGFP mice were imaged using a TCS SP8 confocal laser scanning microscope (Leica Microsystems, Germany). Low‐magnification overview images were acquired with a 10× air objective at 0.75× digital zoom and a line average of 3. For high‐resolution dendritic spine analysis, dendritic segments were imaged with a 63× oil immersion objective at 4× digital zoom. *Z*‐stack images consisting of 15 optical sections at 0.5 μm intervals (total depth: 7 μm) were collected at 1024 × 1024 resolution with a pinhole size of 0.36 Airy units. Apical and basal dendritic branches were imaged for spine analysis. A total of 5 mice per group (*n* = 5) were included for all analyses. All image acquisition and quantitative analyses were performed by investigators blinded to experimental conditions.

#### 2.6.3 PSD95 Expression

PSD95 expression was visualized by immunofluorescence using a high‐affinity anti‐PSD95 antibody. Confocal imaging was performed with a 63× oil immersion objective at 8× digital zoom. The pinhole was set to 0.2 Airy units to enhance optical resolution. Z‐stack images consisting of two optical sections were acquired at 0.25 μm intervals (1024 × 1024 resolution, line averaging = 2). Images were obtained from anatomically matched regions of the CA1 stratum radiatum or dentate gyrus (DG), using identical acquisition settings across all experimental groups.

PSD95 fluorescence intensity was quantified using ImageJ/Fiji. Regions of interest (ROIs) were defined in anatomically matched areas, and mean fluorescence intensity was measured after background correction using the following formula: corrected mean intensity = ROI mean intensity − background mean intensity.

For image preprocessing, all images were converted to 8‐bit format and background‐subtracted using the Subtract Background function in ImageJ/Fiji with a rolling‐ball radius of 30 pixels (Light Background option enabled). Identical processing parameters were applied across all groups to ensure analytical consistency.

The mean value from each animal was used as one biological replicate (*n* = 5 mice/group). All image acquisition and quantitative analyses were performed by investigators blinded to group allocation.

### 2.7 Western Blotting

Hippocampal protein extracts were prepared using RIPA buffer (WB‐0071, Ding Guo, China) supplemented with protease and phosphatase inhibitor cocktails (Beyotime Biotechnology, China). Protein concentrations were determined with a BCA Protein Assay Kit (P0006, Beyotime Biotechnology, China). Equal amounts of protein (10 μg per lane) were mixed with loading buffer, denatured by boiling at 95°C for 5 min, and separated via SDS‐PAGE on 5%–15% polyacrylamide gels. Following electrophoresis, proteins were transferred to polyvinylidene difluoride (PVDF) membranes (ISEQ00010, Millipore).

Membranes were blocked with 5% non‐fat milk in TBST (TBS containing 0.1% Tween‐20) for 1 h at room temperature and then incubated overnight at 4°C with the following primary antibodies: anti‐PSD95 (rabbit, CST, Cat# 3450S, RRID:AB_2292883, 1:1000), anti‐synaptophysin (rabbit, Abcam, Cat#ab32127, RRID:AB_2286949, 1:5000), anti‐CaMKII (rabbit, Selleck, Cat#A5622, RRID:AB_3094758,1:1000), anti‐NMDAR2A (rabbit, Proteintech, Cat# 28525‐1‐AP, RRID:AB_2881163, 1:3000), anti‐NMDAR2B (rabbit, Proteintech, Cat#21920‐1‐AP, RRID:AB_11232223, 1:2000), anti‐LC3 (rabbit, NOVUS, Cat #NB100‐2220, RRID:AB_10003146, 1:1000), anti‐p62 (SQSTM1) (rabbit, Abcam, Cat# ab109012, RRID:AB_10843139, 1:10,000), and anti‐β‐actin (mouse, Proteintech, Cat# 66009‐1‐Ig, RRID:AB_2687938, 1:6000). After washing three times with TBST, membranes were incubated with HRP‐conjugated secondary antibodies for 1 h at room temperature: Goat Anti‐Mouse (1:5000, Proteintech, Cat# SA00001‐1, RRID:AB_2722565) and Goat Anti‐Rabbit (1:5000, Proteintech, Cat# SA00001‐2, RRID:AB_2722564). Protein bands were visualized using an enhanced chemiluminescence (ECL) detection kit (Beyotime, Jiangsu, China), and images were acquired using an automatic chemiluminescence imaging system (Bio‐Rad, Hercules, USA). Band intensities were quantified using ImageJ software and normalized to β‐actin.

### 2.8 Quantitative Real‐Time PCR Analysis

Total RNA was extracted from hippocampal tissues of mice using the Hipure Total RNA Mini Kit (Magen, Guangzhou, China) according to the manufacturer's instructions. RNA concentration and purity were measured using a NanoDrop spectrophotometer (Thermo Fisher Scientific, Waltham, MA, USA). Complementary DNA (cDNA) was synthesized using the TransScript One‐Step gDNA Removal and cDNA Synthesis SuperMix (TransGen Biotech, Beijing, China) following the manufacturer's protocol.

Quantitative real‐time PCR (qPCR) was performed using Hieff qPCR SYBR Green Master Mix (Low Rox Plus) (Yeasen, Shanghai, China) on a Quantagene q225 Real‐Time PCR System (Kubo Technology, Beijing, China). Each 20 μL reaction contained 10 μL SYBR Green Master Mix, 0.4 μL forward primer, 0.4 μL reverse primer, 2 μL cDNA template, and nuclease‐free water. The amplification protocol consisted of an initial denaturation at 95°C for 5 min, followed by 40 cycles of 95°C for 10 s and 60°C for 30 s. A melting curve analysis was performed to verify amplification specificity.

Relative gene expression levels were calculated using the 2^−ΔΔCt^ method and normalized to Gapdh as the internal reference gene. All samples were analyzed in technical triplicates. Target genes analyzed in hippocampal tissues from SSD mice included Iba1, Cx3cr1, and Thy1, depending on experimental grouping.

### 2.9 Metabolomics Study Methods

#### 2.9.1 Chemicals and Reagents

All solvents and reagents used in this study were of LC–MS grade unless otherwise specified. Methanol (MeOH) was obtained from Fisher Scientific (Loughborough, UK), and the internal standard 2‐amino‐3‐(2‐chlorophenyl)‐propionic acid (2‐chloro‐L‐phenylalanine) was purchased from Aladdin (Shanghai, China). Ultrapure water was generated using a Milli‐Q system (Millipore, Bedford, USA).

#### 2.9.2 Equipment

The following laboratory equipment was employed: high‐speed centrifuge (Hunan Xiangyi Experimental Equipment Co. Ltd., Hunan, China), centrifugal vacuum evaporator (Eppendorf China Ltd., Shanghai, China), vortex mixer (Haimen Kylin‐Bell Lab Instruments Co. Ltd., Haimen, China), and 0.22 μm microporous membrane filters (Tianjin Jinteng Experimental Equipment Co. Ltd., Tianjin, China).

#### 2.9.3 Sample Collection and Preparation

Mouse blood was collected into EDTA‐coated vacutainer tubes to prevent coagulation. Plasma was isolated by centrifugation at 3500 × g for 10 min at 4°C, and the supernatant was stored at −80°C until analysis.

For metabolite extraction, 100 μL of plasma was thawed at 4°C and vortexed for 1 min. Next, 400 μL of LC–MS grade methanol was added to precipitate proteins. After vortexing again for 1 min, the mixture was centrifuged at 12,000× *g* for 10 min at 4°C. The supernatant was transferred to a new 2 mL tube and dried using a centrifugal vacuum evaporator under a gentle nitrogen stream.

The dried residue was reconstituted in 150 μL of a 4‐ppm solution of 2‐chloro‐L‐phenylalanine (prepared in 80% methanol–water), followed by 1 min of vortexing. The reconstituted solution was filtered through a 0.22 μm membrane and transferred to LC–MS vials for analysis.

#### 2.9.4 Metabolite Profiling by Liquid Chromatography–Mass Spectrometry (LC–MS/MS)

Metabolomic profiling was conducted using an Agilent 1290 Infinity UHPLC system coupled to a Thermo Fisher Q Exactive hybrid quadrupole–Orbitrap mass spectrometer. Chromatographic separation was achieved on a reversed‐phase C18 column (2.1 mm × 100 mm, 1.8 μm particle size) at a flow rate of 0.3 mL/min. The mobile phases consisted of 0.1% formic acid in water (A) and 0.1% formic acid in acetonitrile (B), with a gradient program as follows: 0–2 min, 5% B; 2–10 min, 5%–95% B; 10–12 min, 95% B; 12–15 min, 5% B.

Mass spectrometric detection was performed in both positive and negative ion modes with the following settings: capillary voltage, 3.5 kV; source temperature, 300°C; and sheath gas flow rate, 45 arbitrary units. Full‐scan MS spectra were acquired over an m/z range of 70–1050 at a resolution of 70,000 (at m/z 200). Data‐dependent acquisition (DDA) mode was used for MS/MS, with collision energy set to 30 eV.

#### 2.9.5 Experimental Repeatability and Statistical Considerations

Each plasma sample was analyzed in triplicate. Metabolites with a coefficient of variation (CV) < 20% and detection rate > 80% across all samples were retained for further analysis.

#### 2.9.6 Identification and Quantification of Differential Metabolites

Significant differential metabolites between experimental groups (Ctrl vs. SSD, Ctrl vs. LSD, and LSD vs. LSD + D30) were identified based on a *p*‐value < 0.05 and fold change (FC) > 1.5. Metabolites were annotated by matching retention times, m/z values, and MS/MS fragmentation spectra to entries in the Human Metabolome Database (HMDB) and METLIN.

Quantification was performed using internal standard calibration. Calibration curves were established using known concentrations, and metabolite levels were determined by comparing peak areas to those of the internal standard. Results were expressed as nanomoles per liter (nmol/L).

#### 2.9.7 Data Processing and Visualization

##### 2.9.7.1 Venn Diagram

A Venn diagram was generated to depict overlapping and unique metabolites among the groups, aiding in the identification of shared and distinct metabolic alterations.

##### 2.9.7.2 Heatmap Analysis

Z‐score normalization and hierarchical clustering were applied to generate heatmaps illustrating relative metabolite abundance across samples.

##### 2.9.7.3 Volcano Plot

Volcano plots were used to visualize the magnitude (log_2_ FC) and significance (−log_10_ *p*‐value) of metabolite changes, highlighting the most differentially expressed features.

##### 2.9.7.4 Pathway Enrichment

Kyoto Encyclopedia of Genes and Genomes (KEGG) pathway enrichment analysis was performed for significantly altered metabolites. Pathways with *p* < 0.05 were considered significantly enriched, providing insight into perturbed biological processes.

### 2.10 Statistical Analysis

All continuous variables were tested for normality using the Shapiro–Wilk test. Normally distributed data are presented as mean ± SEM, whereas non‐normally distributed data are expressed as median with interquartile range [P50 (P25, P75)]. Statistical analyses were performed according to experimental design. For experiments involving four groups, including Ctrl, Ctrl + D30, SSD/LSD, and SSD/LSD + D30, data were analyzed using ordinary two‐way ANOVA, with experimental condition (Ctrl versus SSD/LSD) and treatment (vehicle versus D30) as the two independent factors. The main effects of experimental condition and treatment, as well as their interaction, were evaluated. Where appropriate, Šídák's multiple‐comparisons test was subsequently performed for predefined biologically relevant comparisons, particularly Ctrl versus SSD/LSD and SSD/LSD versus SSD/LSD + D30. For comparisons between two groups, unpaired two‐tailed Student's *t*‐test was used for normally distributed data, and the Mann–Whitney *U* test was applied for non‐normally distributed data. For datasets involving more than two groups but not conforming to a full two‐factor design, one‐way ANOVA with Tukey's post hoc test was used for normally distributed data, whereas the Kruskal–Wallis test with Dunn's post hoc test and Bonferroni correction was used for non‐normally distributed data. To limit inflation of Type I error, post hoc analyses were restricted to biologically relevant, predefined comparisons. All statistical analyses were performed using GraphPad Prism 9.0.0 (GraphPad Software Inc., Boston, MA, USA). All tests were two‐tailed, and *p* < 0.05 was considered statistically significant.

## 3 Results

### 3.1 SSD Disrupts Hippocampal Thy1‐EGFP Expression and Microglial Morphology, Which Are Attenuated by D30 Treatment

To investigate the impact of short‐term sleep deprivation (SSD) on hippocampal neuron–microglia homeostasis and to evaluate the potential protective effects of D30, 12‐month‐old Thy1‐EGFP mice and age‐matched C57BL/6 mice were subjected to a 6‐day SSD protocol (Figure 1A). Thy1‐EGFP mice were used to assess neuronal Thy1‐EGFP expression and microglial morphology, whereas C57BL/6 mice were further employed to examine neuronal integrity and apoptosis through NeuN and cleaved caspase‐3 immunofluorescence analysis. Because the hippocampus is critically involved in learning and memory and is particularly vulnerable to sleep disturbance, subsequent analyses focused on neuronal Thy1‐EGFP expression and microglial alterations within this region. Immunofluorescence staining revealed that SSD markedly reduced Thy1‐EGFP fluorescence and IBA1 immunoreactivity in the dentate gyrus (DG) compared with control mice (Figure 1B–D). Quantitative analysis confirmed a significant decrease in relative Thy1‐EGFP fluorescence intensity and the relative density of IBA1‐positive profiles following SSD, whereas D30 treatment significantly attenuated these reductions (Figure 1C,D). These findings suggest that SSD suppresses hippocampal neuronal Thy1‐EGFP expression and alters IBA1‐labeled microglial profiles, both of which are partially preserved by D30 administration.

To further assess microglial structural remodeling, high‐magnification IBA1 images were subjected to morphological and Sholl‐based analyses (Figure 1E). SSD induced a pronounced simplification of microglial morphology, as reflected by reduced maximum branch length and decreased branch numbers (Figure 1F,G). In contrast, D30‐treated SSD mice displayed a partial restoration of microglial ramification, with branch length and branch number restored toward control levels. These results indicate that D30 mitigates SSD‐induced impairment of microglial morphological complexity.

To further validate whether the reduction in Thy1‐EGFP fluorescence was associated with neuronal loss or apoptosis, we next performed additional analyses in C57BL/6 mice. NeuN and cleaved caspase‐3 immunofluorescence staining in the DG revealed no obvious decrease in NeuN‐positive neurons and no apparent increase in cleaved caspase‐3‐positive neuronal signals following SSD (Figure 1H). Quantitative analysis further confirmed that NeuN‐positive cell density and the proportion of cleaved caspase‐3‐positive NeuN‐positive neurons were not significantly altered among the groups (Figure 1I,J). These results indicate that SSD does not induce overt neuronal loss or caspase‐3‐dependent neuronal apoptosis under the present experimental conditions, suggesting that the observed reduction in Thy1‐EGFP fluorescence is more likely attributable to downregulation of neuronal Thy1 expression and/or reduced Thy1‐EGFP reporter signal intensity rather than neuronal loss.

To explore the molecular basis underlying these cellular alterations, hippocampal mRNA expression of Thy1, Iba1, and Cx3cr1 was examined in 12‐month‐old C57BL/6 mice subjected to the same SSD paradigm (Figure S1B). SSD significantly downregulated Thy1 mRNA expression (Figure S1C), consistent with the observed reduction in neuronal Thy1‐EGFP fluorescence. In parallel, Iba1 and Cx3cr1 transcript levels were also significantly decreased following SSD (Figure S1D,E), suggesting that SSD suppresses microglial marker expression and may disrupt neuron–microglia communication. D30 attenuated the SSD‐induced reduction in Iba1 expression and increased Cx3cr1 expression under SSD, although the condition × treatment interaction for Cx3cr1 was not significant. Notably, these transcriptional changes should be considered when interpreting IBA1‐based microglial profiling, as reduced Iba1 expression may partially contribute to the apparent decrease in IBA1 immunoreactivity, independent of changes in microglial cell number. Thus, IBA1‐based imaging was interpreted as reflecting combined changes in microglial marker expression and morphological remodeling, rather than direct evidence of microglial loss.

Collectively, these results demonstrate that SSD suppresses hippocampal Thy1 expression, reduces IBA1/Cx3cr1‐associated microglial marker expression, and disrupts microglial morphological complexity in middle‐aged mice. Further validation in C57BL/6 mice indicates that these alterations are not accompanied by significant neuronal loss or caspase‐3‐mediated apoptosis. D30 treatment attenuates these SSD‐induced changes, supporting its potential role in preserving hippocampal neuron–microglia homeostasis under sleep deprivation conditions.

### 3.2 SSD Reduces Cx3cr1‐EGFP and IBA1‐Labeled Microglial Complexity in the Hippocampus

**FIGURE 2:**
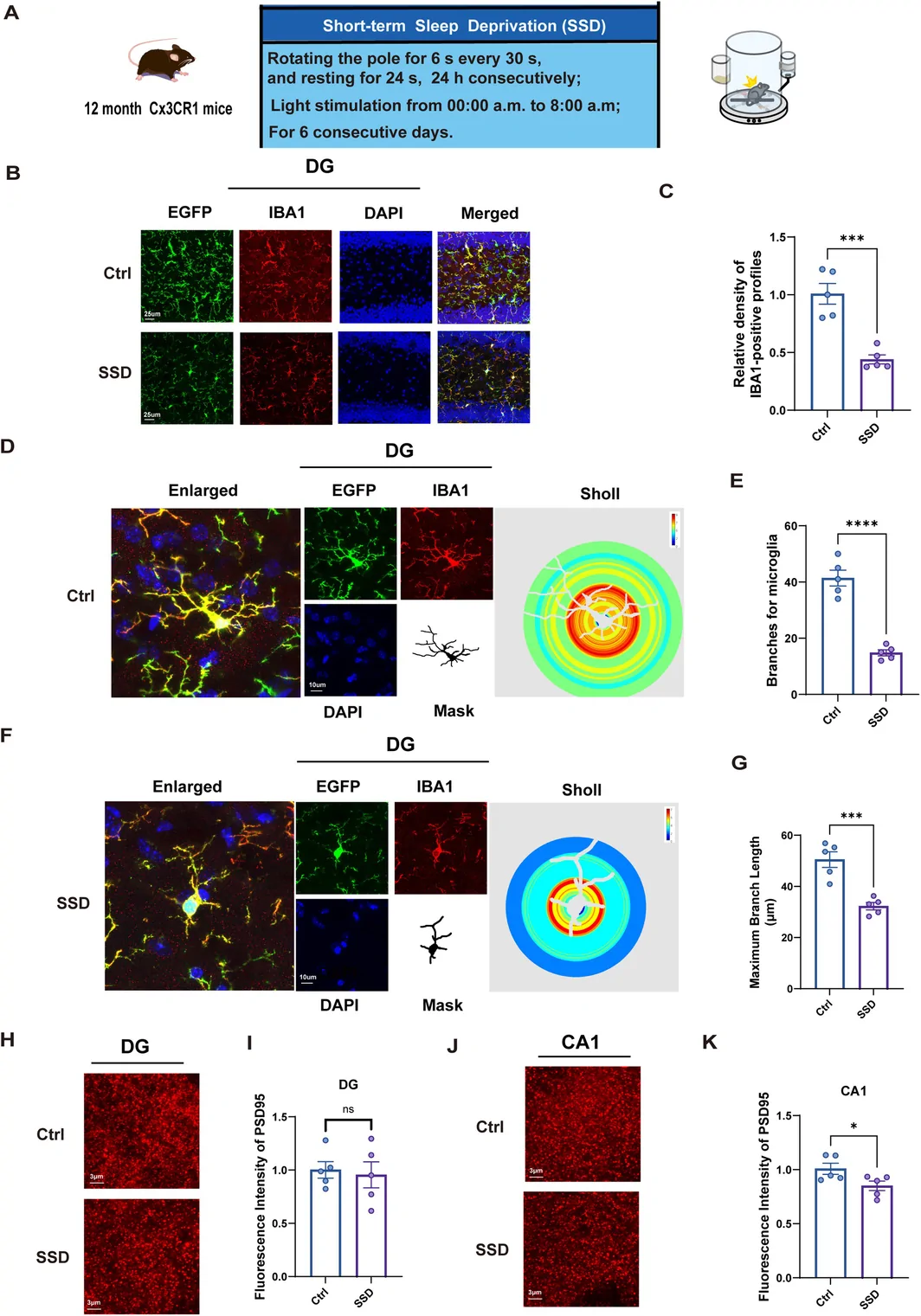
SSD impairs microglial marker‐associated profiles, microglial complexity, and synaptic marker expression in the hippocampus of Cx3CR1‐EGFP mice. (A) Schematic of the SSD experimental protocol. (B) Representative immunofluorescence images showing IBA1‐positive microglia (red) in the dentate gyrus (DG) region of control and SSD‐treated 12‐month‐old Cx3CR1‐EGFP mice. (C) Quantification of IBA1‐positive microglial density in the DG. (D, F) High‐magnification images of microglia from control (D) and SSD‐treated (F) mice. (E, G) Sholl analysis of microglial morphology showing (E) number of branches and (G) maximum branch length. (H, I) Representative images (H) and quantification (I) of PSD95 expression in the DG. (J, K) Representative images (J) and quantification (K) of PSD95 expression in the CA1 region. Data are presented as mean ± SEM (*n* = 5 animals per group). Statistical significance was determined by unpaired two‐tailed Student's *t*‐test. ns, not significant; *p < 0.05, ***p < 0.001, ****p < 0.0001.

Building on our findings in Thy1‐EGFP and C57BL/6J mice, which showed that SSD reduces Thy1‐EGFP fluorescence and decreases hippocampal Iba1 and Cx3cr1 mRNA expression, we next employed 12‐month‐old Cx3CR1‐EGFP transgenic mice to further characterize SSD‐induced microglial alterations. This reporter model enables direct visualization of microglial distribution and morphology (Figures 2A and S2A). Cx3CR1 is a chemokine receptor predominantly expressed by microglia in the central nervous system and plays an important role in neuron–microglia communication and immune homeostasis.

Following 6 days of SSD, we observed a significant reduction in EGFP fluorescence intensity throughout the hippocampus, alongside a concomitant decrease in Cx3CR1 mRNA levels (Figure S1E), suggesting reduced Cx3cr1 transcriptional activity or altered reporter signal output. Quantification revealed a substantial reduction in EGFP‐positive microglial profiles and marker‐associated signals in the DG and CA1 regions of SSD‐treated mice. The remaining EGFP‐positive microglial profiles exhibited lower fluorescence intensity and markedly simplified branching patterns. Co‐immunostaining with IBA1 confirmed substantial colocalization with EGFP and further indicated a significant decline in IBA1‐positive microglial profiles after SSD (Figures 2B,C and S2B,C). Notably, these changes likely reflect combined alterations in microglial marker expression and morphological remodeling rather than direct evidence of microglial loss.

High‐resolution confocal imaging combined with Sholl analysis revealed that microglia from SSD‐treated mice exhibited fewer and shorter branches compared with controls. These morphological alterations were consistently observed in both the dentate gyrus (DG) and CA1 subregions (Figures 2D–G and S2D–G). Furthermore, three‐dimensional reconstructions of microglial arbors acquired under identical imaging conditions (Figure S3) further highlighted the reduced structural complexity of microglia following SSD exposure.

To determine whether SSD also impacts synaptic integrity beyond Thy1‐EGFP downregulation, we assessed the expression of the postsynaptic density protein PSD95. In the DG, PSD95 levels were largely unaltered following SSD (Figure 2H,I); however, a significant reduction was detected in the CA1 region (Figure 2J,K). These findings indicate a region‐specific vulnerability of hippocampal synapses to sleep deprivation, consistent with prior studies [22, 40, 41, 42].

Collectively, these findings suggest that SSD induces pronounced microglial marker and morphological alterations in middle‐aged mice, whereas its impact on PSD95 expression appears modest and region‐dependent.

### 3.3 SSD Induces Marked Alterations in Plasma Metabolites in 12‐Month‐Old Thy1‐EGFP Mice

To evaluate the systemic metabolic impact of SSD, untargeted plasma metabolomic profiling was performed in 12‐month‐old Thy1‐EGFP mice. Hierarchical clustering analysis revealed a clear separation between SSD and control groups (Figure S4A), indicating a widespread shift in circulating metabolic signatures. Consistently, volcano plot analysis identified a substantial number of significantly upregulated and downregulated metabolites associated with SSD exposure (Figure S4B).

Among these, 3‐hydroxyechinenone—a carotenoid derivative with reported antioxidant activity—was markedly decreased following SSD, potentially indicating a reduction in circulating antioxidant‐related metabolites. In contrast, metabolites such as 2‐furanmethanol and 2‐pentanone, which have been reported in association with lipid oxidation or xenobiotic metabolism, were significantly elevated.

To further elucidate the functional implications of these changes, KEGG pathway enrichment analysis was conducted (Figure S4C). SSD‐induced alterations were primarily enriched in pathways related to energy metabolism, including oxidative phosphorylation and the tricarboxylic acid (TCA) cycle, as well as amino acid metabolism (e.g., alanine, aspartate, and glutamate metabolism) and hormonal regulation (e.g., glucagon signaling), suggesting disruption of systemic bioenergetic and metabolic homeostasis.

In addition, KEGG‐based network analysis identified several central metabolic nodes affected by SSD (Figure S4D). These included intermediates such as citric acid and β‐D‐glucose‐6‐phosphate, reflecting perturbations in mitochondrial energy metabolism and glucose utilization. Further perturbations were observed in branched‐chain amino acid biosynthesis (valine, leucine, isoleucine), glycine/serine/threonine metabolism, and one‐carbon metabolism, supported by altered levels of L‐valine, carbamoyl phosphate, flavin mononucleotide, and pyrophosphate.

Collectively, these findings demonstrate that SSD induces significant and multi‐dimensional alterations in plasma metabolite composition in 12‐month‐old mice, underscoring the metabolic vulnerability of middle‐aged mice to acute sleep disruption.

### 3.4 D30 Ameliorates Behavioral and Cognitive Task Impairments Induced by LSD in 12‐Month‐Old Thy1‐EGFP Mice

**FIGURE 3:**
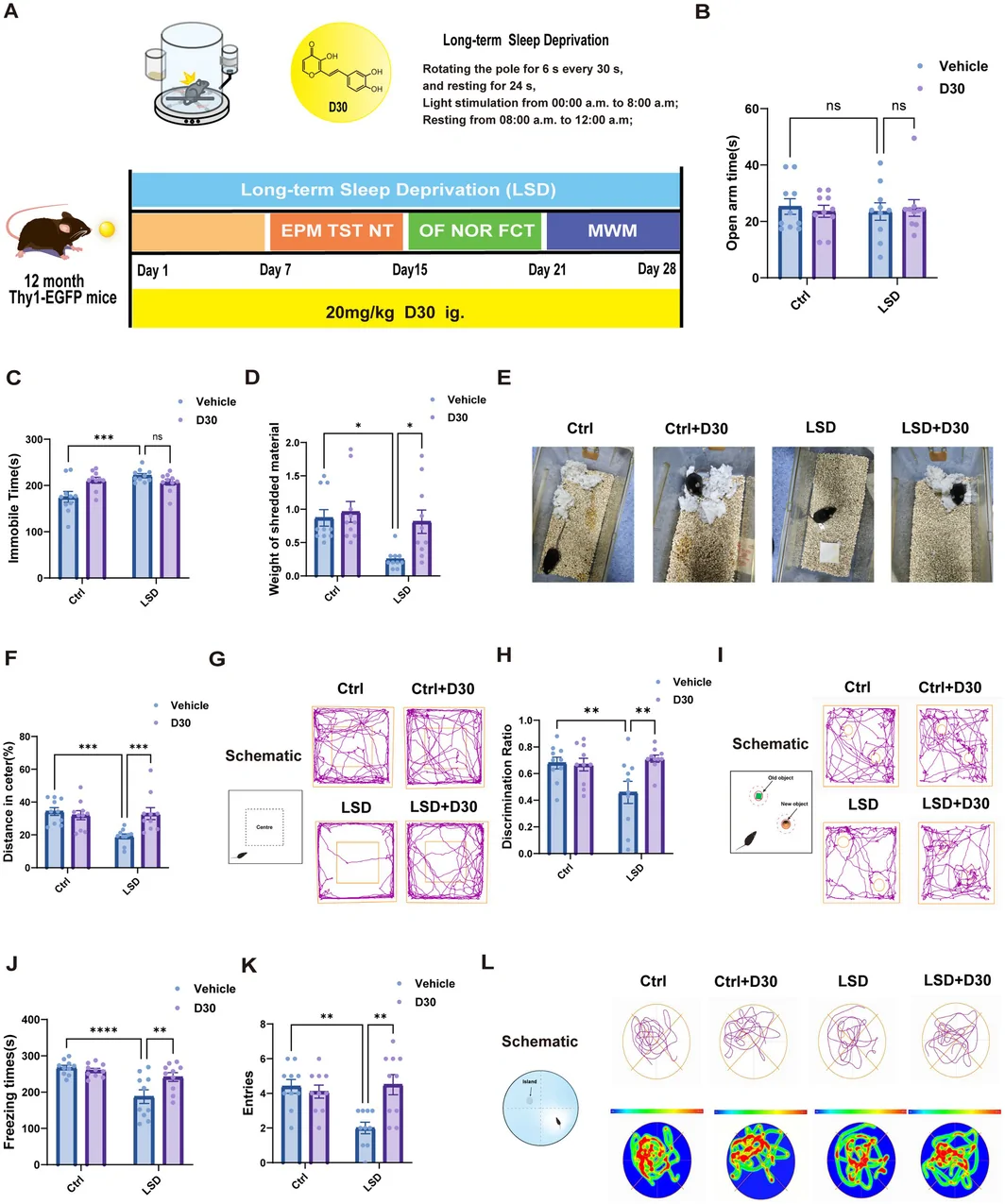
D30 treatment mitigates cognitive and behavioral deficits induced by LSD in 12‐month‐old Thy1‐EGFP mice. (A) Schematic representation of the LSD protocol and D30 administration regimen in 12‐month‐old Thy1‐EGFP mice (20 mg/kg, i.g. daily for 28 days). (B) Elevated plus maze (EPM) test showed no significant differences in open arm time across groups, indicating no significant effect of LSD on EPM performance under the present testing conditions. (C) Tail suspension test (TST) indicated increased immobility time in LSD‐treated mice, consistent with depression‐like behavioral alterations; D30 treatment did not significantly reverse this effect under the present experimental conditions. (D) Nesting test (NT) revealed significantly impaired nest‐building behavior in LSD mice, as reflected by reduced weight of shredded nesting material, which was partially rescued by D30 treatment. (E) Representative nest images corresponding to the groups shown in (D). (F) Open field (OF) test demonstrated that LSD reduced the percentage of distance traveled in the center zone, suggesting increased center‐zone avoidance and OF‐based anxiety‐like behavior; this effect was partially ameliorated by D30 treatment. (G) Representative locomotor traces from the OF test. (H) Novel object recognition (NOR) test showed reduced discrimination ratio in the LSD group, reflecting impaired recognition memory; D30 treatment significantly improved this parameter. (I) Representative exploratory trajectories from the NOR test. (J) Fear conditioning test (FCT) demonstrated reduced freezing behavior in LSD mice, consistent with compromised associative memory; D30 treatment significantly restored freezing behavior. (K) In the Morris water maze (MWM), LSD led to reduced platform crossings, indicative of impaired spatial memory, which was significantly improved by D30 treatment. (L) Representative swim paths and heatmaps from the MWM test on the probe day. Data are presented as mean ± SEM, with each dot representing one animal (*n* = 10 mice per group). Statistical significance was assessed using ordinary two‐way ANOVA with experimental condition (Ctrl vs. LSD) and treatment (vehicle vs. D30) as factors, followed by Šídák's multiple‐comparisons test. For (B), neither the main effect of experimental condition (*F*(1, 36) = 0.01195, *p* = 0.9136), the main effect of treatment (*F*(1, 36) = 0.008446, *p* = 0.9273), nor their interaction (*F*(1, 36) = 0.2865, *p* = 0.5958) was significant. For (C), the main effect of experimental condition was significant (*F*(1, 36) = 7.324, *p* = 0.0103), whereas the main effect of treatment was not (*F*(1, 36) = 2.001, *p* = 0.1658); the interaction was significant (*F*(1, 36) = 10.51, *p* = 0.0026). For (D), significant main effects of experimental condition (*F*(1, 36) = 8.123, *p* = 0.0072) and treatment (*F*(1, 36) = 5.855, *p* = 0.0207) were detected, whereas their interaction was not significant (*F*(1, 36) = 3.068, *p* = 0.0884). For (F), significant main effects of experimental condition (*F*(1, 36) = 6.962, *p* = 0.0122) and treatment (*F*(1, 36) = 4.654, *p* = 0.0377), together with a significant experimental condition × treatment interaction (*F*(1, 36) = 9.031, *p* = 0.0048), were detected. For (H), the main effect of treatment was significant (*F*(1, 36) = 4.978, *p* = 0.0320), whereas the main effect of experimental condition was not significant (*F*(1, 36) = 2.654, *p* = 0.1120); the experimental condition × treatment interaction was significant (*F*(1, 36) = 5.962, *p* = 0.0197). For (J), significant main effects of experimental condition (*F*(1, 36) = 16.77, *p* = 0.0002) and treatment (*F*(1, 36) = 4.222, *p* = 0.0472), together with a significant experimental condition × treatment interaction (*F*(1, 36) = 7.432, *p* = 0.0098), were detected. For (K), significant main effects of experimental condition (*F*(1, 36) = 5.310, *p* = 0.0271) and treatment (*F*(1, 36) = 6.425, *p* = 0.0157), together with a significant experimental condition × treatment interaction (*F*(1, 36) = 10.41, *p* = 0.0027), were detected. ns, not significant; *p < 0.05, **p < 0.01, ***p < 0.001, and ****p < 0.0001 for the indicated post hoc comparisons.

To investigate the impact of LSD and the protective effect of D30 in 12‐month‐old Thy1‐EGFP mice, a battery of behavioral assessments was conducted after day 7 during the 28‐day LSD paradigm. D30 was administered daily throughout the 28‐day period to evaluate its efficacy in alleviating behavioral impairments induced by chronic sleep loss. To address potential confounding effects related to general health status, body weight and food intake were monitored during the experimental period. Body weight remained generally stable across groups, and no obvious treatment‐related body weight loss was observed. Daily food intake also showed no consistent reduction associated with either LSD exposure or D30 treatment, suggesting that the behavioral outcomes were unlikely to be primarily driven by systemic deterioration or altered feeding behavior (Figures 3A and S5A,B).

In the elevated plus maze test (EPM), performed during the second week of LSD, no significant differences in open arm time were observed among the groups, indicating that LSD did not significantly alter EPM performance under this testing condition (Figure 3B). In the tail suspension test (TST), LSD‐treated mice exhibited significantly increased immobility time compared with control mice, suggesting depression‐like behavioral alterations. However, D30 treatment did not significantly improve this parameter under the present experimental conditions (Figure 3C).

In contrast, the nesting test (NT) revealed pronounced impairments in innate nesting behavior after LSD exposure, as reflected by reduced weight of shredded nesting material. Notably, D30 treatment significantly improved nest‐building performance, as further supported by representative nest images (Figure 3D,E). Given that nesting behavior can also reflect general well‐being, the absence of overt body weight loss or reduced food intake further supports that the nesting impairment was not simply attributable to poor general condition.

In the open field (OF) test conducted during the third week of LSD, LSD‐treated mice showed a significantly reduced percentage of distance traveled in the center zone compared with control mice, suggesting increased center‐zone avoidance and OF‐based anxiety‐like behavior. D30 administration effectively mitigated this behavioral deficit (Figure 3F), as further illustrated by representative locomotor traces (Figure 3G). Importantly, total distance traveled in the OF test did not differ significantly among groups, indicating that the altered center‐zone behavior was not attributable to impaired general locomotor activity (Figure S5C).

Recognition memory was evaluated using the novel object recognition (NOR) test. LSD markedly reduced the discrimination ratio, indicating impaired recognition memory. D30 treatment significantly improved this parameter, suggesting partial protection against LSD‐induced recognition memory impairment (Figure 3H,I). In parallel, total exploration time during the NOR test was comparable among groups, suggesting that the differences in novel object exploration were not driven by reduced overall exploratory activity or motivation (Figure S5D).

The fear conditioning test further showed that LSD impaired associative memory, as evidenced by reduced freezing behavior during the testing phase. Mice receiving D30 during LSD exposure displayed significantly improved freezing responses, indicating that D30 mitigated LSD‐induced associative memory deficits (Figure 3J).

In the Morris water maze (MWM) test, the number of platform crossings during the probe trial was significantly reduced in the LSD group, indicating impaired spatial learning and memory. D30 treatment significantly increased platform crossings (Figure 3K), while representative swimming trajectories and heatmaps further demonstrated improved spatial search behavior after D30 administration (Figure 3L). Notably, LSD had no significant main effect on swimming speed or total swimming distance, and no condition × treatment interaction was detected. Although D30 showed significant overall main effects on both parameters, pairwise comparisons between the LSD and LSD+D30 groups were not significant (Figure S5E,F). Thus, reduced motor function or swimming ability was unlikely to account for the LSD‐induced spatial memory impairment (Figure S5E,F).

Collectively, these behavioral assessments demonstrate that LSD induces pronounced impairments in depression‐like behavior, innate nesting behavior, OF‐based center‐zone behavior, recognition memory, associative memory, and spatial learning and memory in 12‐month‐old Thy1‐EGFP mice, and that D30 treatment partially ameliorates many of these behavioral deficits.

### 3.5 LSD Alters Hippocampal Thy1‐EGFP Expression and Glial Morphology, Which Are Attenuated by D30 Treatment

**FIGURE 4:**
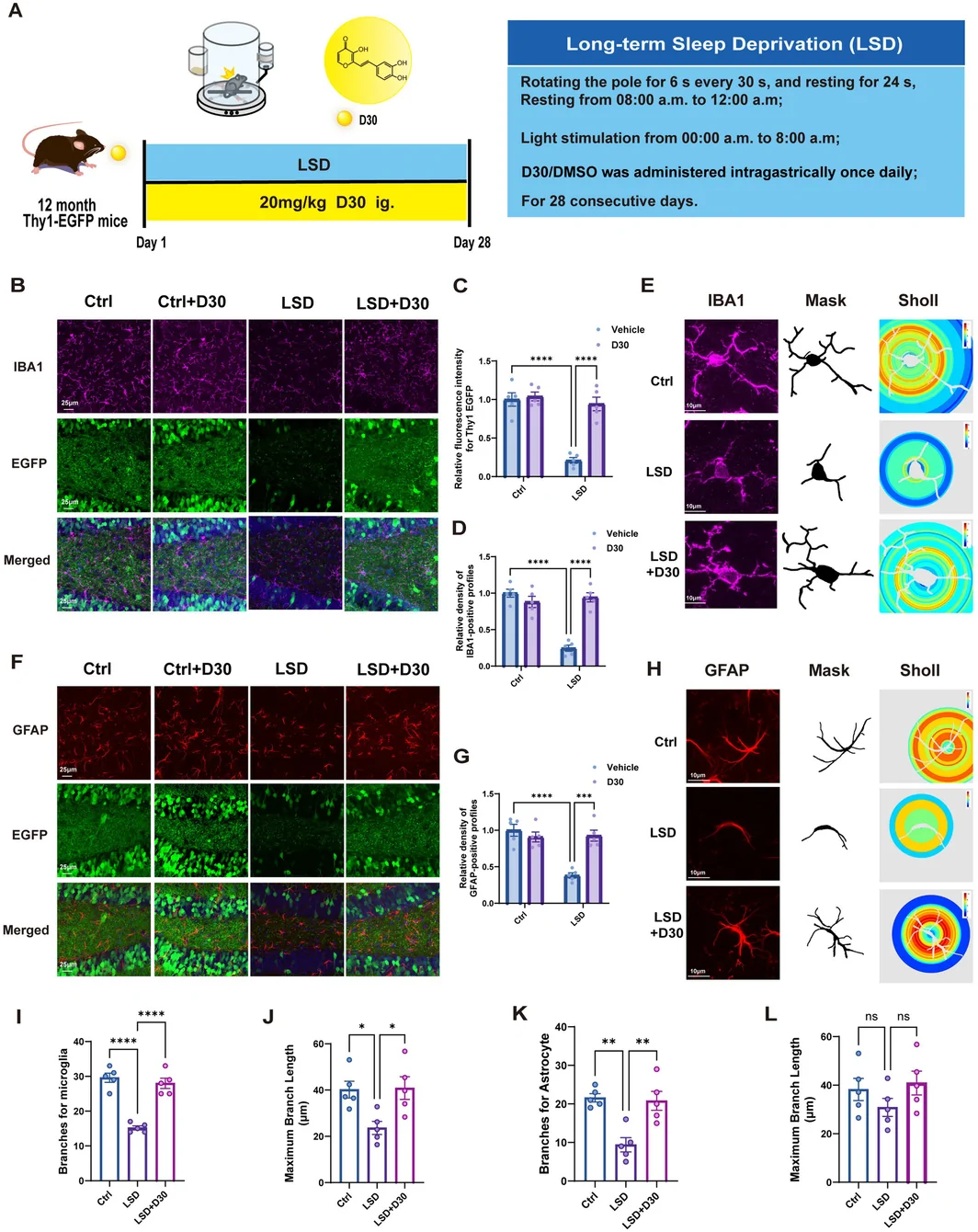
D30 attenuates LSD‐induced reductions in hippocampal Thy1‐EGFP and glial marker‐associated signals and preserves glial cell morphology. (A) Experimental schematic of the 28‐day LSD model and D30 administration in 12‐month‐old Thy1‐EGFP mice. (B) Representative immunofluorescence images of hippocampal IBA1‐positive microglial profiles (magenta) and Thy1‐EGFP‐positive neurons (green) in each group. (C) Quantification of Thy1‐EGFP fluorescence intensity. (D) Quantification of IBA1‐positive microglial profile‐associated signals. (E) Representative high‐magnification images, binary masks, and Sholl analysis heatmaps of IBA1‐positive microglia, illustrating reduced branching following LSD and partial rescue by D30. (F) Representative images of GFAP‐positive astrocytic profiles (red) and Thy1‐EGFP‐positive neurons (green) in the hippocampus. (G) Quantification of the relative density of GFAP‐positive astrocytic profiles. (H) Representative high‐magnification images, binary masks, and Sholl analysis heatmaps of GFAP‐positive astrocytes. (I, J) Quantification of the number of microglial branches (I) and maximum branch length (J) from panel E. (K, L) Quantification of the number of astrocytic branches (K) and maximum branch length (L) from panel H. Data are presented as mean ± SEM, with each dot representing one mouse (*n* = 5 mice per group). For C, D, and G, statistical significance was assessed using ordinary two‐way ANOVA with experimental condition (Ctrl versus LSD) and treatment (vehicle versus D30) as factors, followed by Šídák's multiple‐comparisons test. For C, the main effects of experimental condition [*F*(1, 16) = 39.47, *p* < 0.0001] and treatment [*F*(1, 16) = 29.91, *p* < 0.0001], as well as their interaction [*F*(1, 16) = 24.00, *p* = 0.0002], were significant. For D, the main effects of experimental condition [*F*(1, 16) = 32.79, *p* < 0.0001] and treatment [*F*(1, 16) = 22.80, *p* = 0.0002], as well as their interaction [*F*(1, 16) = 45.63, *p* < 0.0001], were significant. For G, the main effects of experimental condition [*F*(1, 16) = 19.87, *p* = 0.0004] and treatment [*F*(1, 16) = 11.38, *p* = 0.0039], as well as their interaction [*F*(1, 16) = 22.47, *p* = 0.0002], were significant. For the three‐group morphological analyses in I–L, statistical significance was assessed using ordinary one‐way ANOVA with Tukey's post hoc test. ns, not significant; *p < 0.05, **p < 0.01, ***p < 0.001, and ****p < 0.0001.

To investigate the impact of LSD on hippocampal neuronal integrity and glial cell homeostasis, we examined Thy1‐EGFP expression and glial markers in 12‐month‐old Thy1‐EGFP mice subjected to a 28‐day LSD protocol with or without concurrent D30 treatment (Figure 4A).

Consistent with the hippocampal findings, LSD markedly reduced Thy1‐EGFP fluorescence intensity in Thy1‐EGFP‐expressing neuronal populations, whereas D30 treatment effectively alleviated this reduction and preserved EGFP signal intensity to levels comparable to those observed in control mice (Figure S6A,B). Importantly, immunofluorescence staining for NeuN and cleaved caspase‐3 revealed no apparent changes in neuronal density or apoptotic activation in either the CA1 region or dentate gyrus following LSD (Figure S6C–E), indicating that the LSD‐induced attenuation of Thy1‐EGFP signal is unlikely to result from overt neuronal loss or caspase‐3‐dependent neuronal apoptosis.

Immunofluorescence analysis demonstrated a significant reduction in both Thy1‐EGFP fluorescence intensity and the relative density of IBA1‐positive microglial profiles in the hippocampus following LSD, which was effectively alleviated by D30 treatment (Figure 4B–D). Given that IBA1 expression may itself be altered under sleep deprivation conditions, IBA1‐based quantification was interpreted as reflecting changes in IBA1 expression levels and microglial morphological profiles rather than direct evidence of microglial cell loss. Sholl analysis of microglial morphology further demonstrated that LSD significantly reduced both the number of microglial branches and the maximum branch length, indicative of reduced microglial morphological complexity. These structural alterations were attenuated by D30 treatment (Figure 4E,I,J).

LSD also resulted in a significant reduction in hippocampal GFAP immunofluorescence intensity (Figure 4F,G), suggesting altered astrocytic marker expression rather than definitive loss of astrocytes. Sholl analysis revealed that LSD significantly reduced the number of GFAP‐positive astrocytic branches, although it did not significantly affect the maximum branch length (Figure 4H,K,L). Notably, D30 treatment preserved the number of astrocytic branches but did not significantly alter the maximum branch length.

Overall, these data indicate that LSD reduces Thy1‐EGFP expression and alters both microglial and astrocytic structural features in the hippocampus, with partial reversal observed following D30 administration.

### 3.6 D30 Treatment Preserves Hippocampal Thy1‐EGFP Expression, Dendritic Spine Integrity, Synaptic Protein Expression, and Autophagy‐Related Markers Under LSD

To further evaluate the structural and molecular underpinnings of cognitive impairment induced by sleep deprivation, we examined changes in Thy1‐EGFP fluorescence, dendritic spine morphology, synaptic proteins, and autophagy‐related markers in the hippocampus of 12‐month‐old Thy1‐EGFP mice following SSD or LSD, with or without concurrent D30 treatment in the LSD paradigm.

**FIGURE 5:**
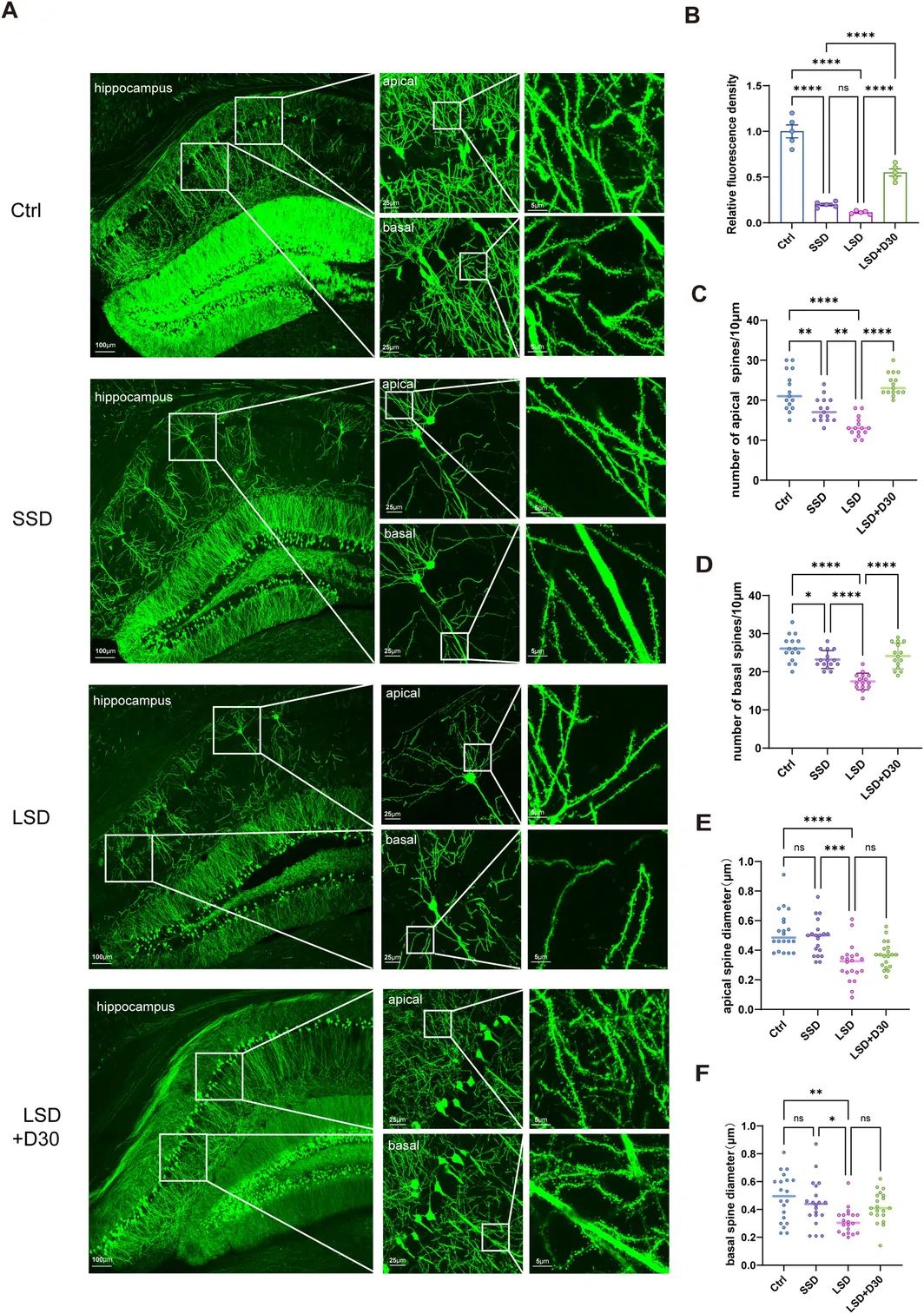
Sleep deprivation reduces Thy1‐EGFP expression, dendritic spine density, and spine diameter in hippocampal CA1 pyramidal neurons, which are largely preserved by D30 treatment. (A) Representative fluorescence images acquired using a 10× objective with a 20 μm *z*‐stack projection. Both short‐term sleep deprivation (SSD, 6 days) and long‐term sleep deprivation (LSD, 28 days) decreased overall Thy1‐EGFP signal intensity across hippocampal CA1 pyramidal neurons, whereas D30 treatment partially mitigated the LSD‐induced reduction. (B) Quantification of Thy1‐EGFP fluorescence intensity across groups (*n* = 5 animals per group). (C, D) Quantification of dendritic spine density in apical (C) and basal (D) dendrites of CA1 pyramidal neurons. Both SSD and LSD significantly reduced spine density, with LSD producing a more pronounced reduction; this effect was attenuated by D30 treatment under the LSD condition. (E, F) Analysis of spine diameter in apical (E) and basal (F) dendrites. LSD, but not SSD, significantly decreased spine diameter, whereas D30 treatment showed no statistically significant rescue of spine diameter. Data are presented as mean ± SEM. ns, not significant; *p < 0.05, **p < 0.01, ***p < 0.001, ****p < 0.0001.

Representative fluorescence imaging showed that both SSD and LSD markedly reduced Thy1‐EGFP signal intensity in hippocampal CA1 pyramidal neurons, with LSD producing a more pronounced reduction. D30 treatment significantly alleviated the LSD‐induced decrease in Thy1‐EGFP fluorescence intensity, although the signal was not fully restored to the level observed in control mice (Figure 5A,B).

To further assess synaptic structural alterations, we quantified dendritic spine density and morphology in CA1 neurons. Both SSD and LSD led to significant reductions in spine density in apical and basal dendrites, with LSD inducing more profound deficits (Figure 5C,D). Importantly, D30 treatment effectively prevented LSD‐induced spine loss and partially preserved dendritic spine density, indicating a protective effect on synaptic structural integrity.

Analysis of spine diameter revealed that LSD, but not SSD, significantly decreased spine head size in both apical and basal compartments (Figure 5E,F), indicating that prolonged sleep deprivation compromises not only the number of spines but also their structural maturation and potentially synaptic efficacy. Although D30 treatment showed a trend toward increasing spine diameter after LSD, this effect did not reach statistical significance, suggesting that D30 primarily preserves synaptic quantity rather than fully restoring spine morphological maturation.

We next examined synaptic protein expression and autophagy‐related markers under the LSD condition. Immunofluorescence staining revealed a marked reduction in PSD95 expression in both the dentate gyrus (DG) and CA1 regions of LSD‐treated mice, suggesting impaired synaptic protein expression (Figure S7A–C). D30 treatment substantially preserved PSD95 density in both subregions, supporting its protective effect on a key postsynaptic component.

Western blot analysis of hippocampal tissue further supported these findings. LSD exposure significantly decreased the expression of key synaptic proteins, including PSD95, NMDAR2A, NMDAR2B, synaptophysin, and CaMKII, whereas D30 treatment largely preserved their levels (Figure S7D–I). In addition, LSD increased the LC3‐II/LC3‐I ratio and reduced p62 levels, indicating altered autophagy‐related responses in the hippocampus. D30 treatment attenuated these LSD‐induced changes in LC3‐II/LC3‐I and p62 (Figure S7J,K). These results support the notion that LSD disrupts synaptic integrity and autophagy‐related processes, which can be mitigated by D30.

Together, these findings demonstrate that sleep deprivation compromises Thy1‐EGFP expression, dendritic spine integrity, synaptic protein expression, and autophagy‐related marker profiles in the hippocampus, with more severe impairments induced by prolonged deprivation. D30 treatment significantly mitigates the detrimental effects of LSD on Thy1‐EGFP signal, spine density, synaptic protein expression, and autophagy‐related markers, but has limited efficacy in restoring spine diameter.

### 3.7 LSD Induces Systemic Metabolic Dysregulation in 12‐Month‐Old Mice, Which Is Partially Reversed by D30 Treatment

**FIGURE 6:**
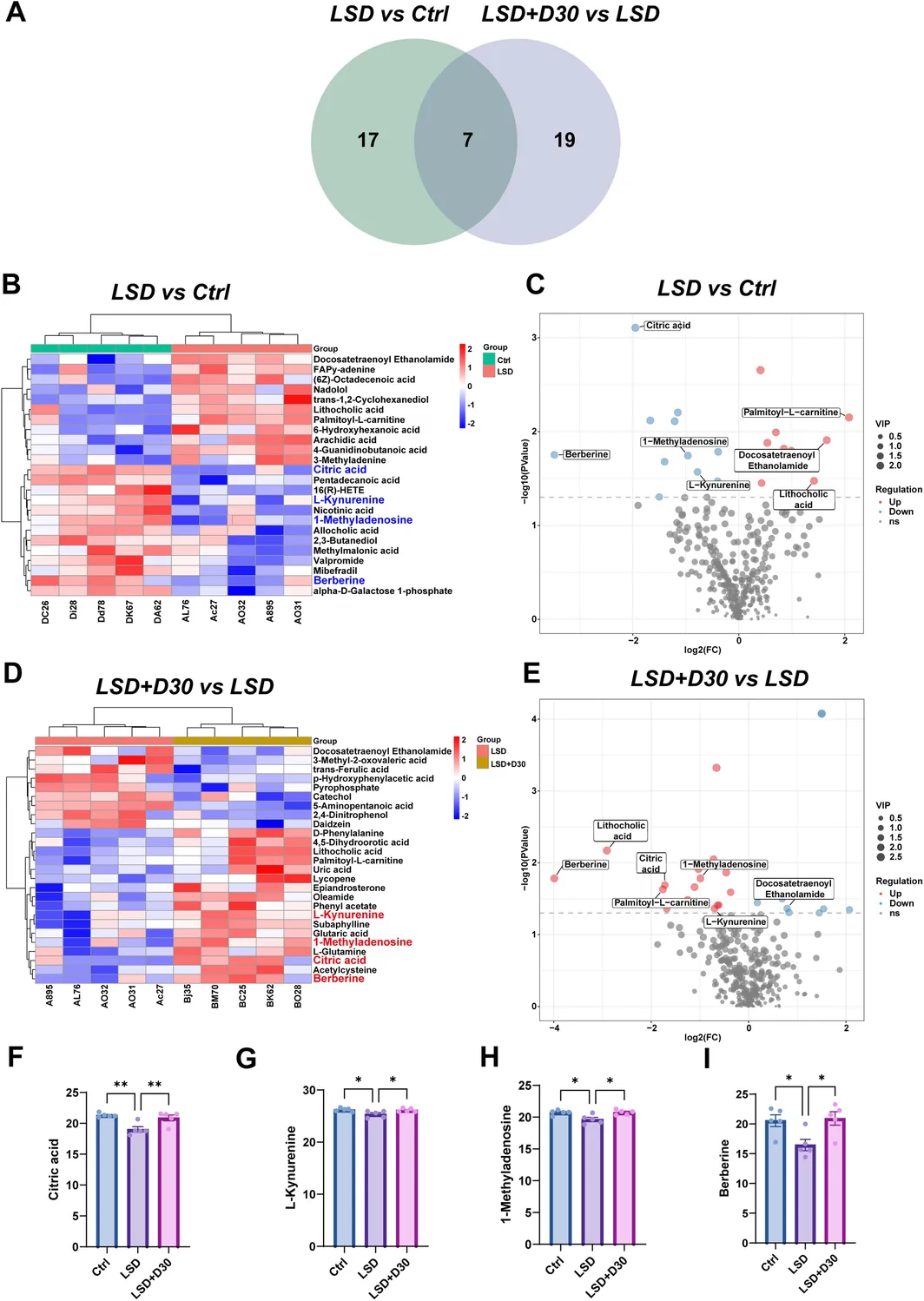
Metabolomic analysis of middle‐aged mice following LSD and D30 treatment. (A) Metabolomic analysis of 12‐month mice following LSD and D30 treatment. (B) Heatmap showing hierarchical clustering of 24 significantly altered metabolites between LSD and Ctrl groups, highlighting widespread metabolic disruptions induced by LSD. (C) Volcano plot for LSD vs. Ctrl, showing significant metabolic shifts induced by LSD. (D) Heatmap of 26 differentially expressed metabolites between LSD + D30 and LSD groups, indicating significant normalization of metabolic profiles following D30 treatment. (E) Volcano plot for LSD + D30 versus LSD, showing significant metabolic shifts induced by D30 on LSD mice. (F–I) Relative abundance of representative differential metabolites: Citric acid (F), L‐kynurenine (G), 1‐methyladenosine (H), and berberine (I), illustrating the effects of LSD and D30 treatment. Data are expressed as mean ± SEM (*n* = 5 animals per group). Statistical significance was determined by one‐way ANOVA followed by post hoc multiple‐comparison tests. ns, not significant; *p < 0.05, **p < 0.01.

To elucidate the systemic metabolic impact of LSD and the potential regulatory effects of D30, we conducted untargeted plasma metabolomic profiling in 12‐month‐old mice. A Venn diagram comparison (Figure 6A) identified seven overlapping differential metabolites between the LSD versus Ctrl and LSD+D30 versus LSD groups, highlighting key metabolic markers that were both disrupted by LSD and modulated by D30 intervention.

Hierarchical clustering revealed 24 significantly altered plasma metabolites in LSD mice compared to controls (Figure 6B), indicating widespread metabolic perturbations involving amino acid metabolism, energy production, and lipid turnover. In contrast, 26 metabolites were differentially regulated in the LSD+D30 versus LSD comparison (Figure 6D), many of which trended toward normalization, suggesting partial restoration of metabolic homeostasis by D30.

Volcano plots further illustrated the extent of metabolic dysregulation. LSD caused significant shifts in plasma metabolite profiles (Figure 6C), while D30 treatment markedly reversed several of these changes (Figure 6E). Among them, four representative metabolites exemplify key nodes of LSD‐induced metabolic disruption and D30‐mediated recovery:

Citric acid (Figure 6F), a central TCA cycle intermediate, was significantly reduced in LSD mice, consistent with impaired mitochondrial function, and was restored by D30 treatment.

L‐kynurenine (Figure 6G), a neuroactive tryptophan metabolite involved in neuroimmune regulation, was diminished following LSD and normalized by D30.

1‐methyladenosine (Figure 6H), an RNA methylation byproduct linked to cellular stress responses, was reduced in LSD mice and partially restored by D30, indicating stress alleviation.

Berberine (Figure 6I), a compound with known AMPK‐activating properties, was reduced by LSD and elevated by D30 treatment, suggesting protective metabolic modulation.

KEGG pathway enrichment analysis supported these findings (Figure S8A,B). LSD significantly disrupted pathways associated with mitochondrial energy metabolism (e.g., the TCA cycle), AMPK signaling, and amino acid metabolism—especially pathways involving alanine, aspartate, and glutamate (Figure S8A). These processes are essential for neurotransmitter cycling, synaptic transmission, and energy homeostasis. D30 treatment modulated the same pathways (Figure S8B), improving fatty acid degradation and restoring glutamatergic and GABAergic signaling. These effects are consistent with the molecular improvements observed in synaptic markers such as PSD95 and NMDAR (Figure S7), both reduced by LSD and preserved by D30.

Collectively, these results demonstrate that LSD induces profound systemic metabolic stress, characterized by mitochondrial dysfunction, neurotransmitter imbalance, and altered lipid and amino acid metabolism. D30 treatment partially reversed these alterations by restoring key metabolites and rebalancing critical metabolic pathways, thereby supporting its role in mitigating sleep deprivation‐induced metabolic and neurological impairments.

## 4 Discussion

### 4.1 Impact on Glial Function: Comparison of SSD and LSD

Our results show that both SSD and LSD lead to glial suppression, but the effects of LSD are more pronounced and enduring. Among glial populations, microglia appear to be the most prominently affected cell type under sleep deprivation. The effects of LSD on microglia were particularly prominent in 12‐month‐old mice. The LSD‐induced reduction in microglial branch number and branch length resembles morphological features commonly associated with senescent microglia [43], suggesting that chronic sleep deprivation may accelerate age‐related microglial functional decline rather than induce classical inflammatory activation. This is consistent with previous reports showing that microglial density decreases after SD in aged mice, whereas microglial activation or proliferation has been observed in younger mice, highlighting age‐dependent heterogeneity in microglial responses to sleep loss.

In addition, we found that SD significantly reduced the expression of Cx3CR1 and IBA1 in microglia that remained after SD exposure, indicative of suppressed microglial activity rather than overt microglial loss or activation [44]. Because IBA1 and Cx3CR1 are involved in microglial motility, surveillance, and neuron–microglia communication, their downregulation may reflect impaired microglial homeostatic function. At the same time, reduced marker expression may influence the apparent detection of microglial profiles and should therefore be considered when interpreting changes in microglial morphology and signal intensity. Interestingly, D30 treatment largely preserved microglial morphological complexity and attenuated the SD‐induced reduction of IBA1 and Cx3cr1 expression, suggesting its neuroprotective effects in maintaining glial homeostasis under chronic sleep deprivation.

Astrocytic changes followed a similar pattern, with LSD leading to a reduction in GFAP expression and altered astrocytic cytoskeletal morphology. Because astrocytic structure is closely coupled to astrocytic function, these findings suggest that LSD may impair astrocyte‐mediated neuronal support, metabolic regulation, and neurotoxic clearance [45]. Notably, D30 treatment mitigated the decrease in GFAP expression levels and GFAP branching, which may contribute to the neuroprotective effects observed in this study. Together, these findings suggest that chronic sleep deprivation disrupts glial homeostasis through both microglial surveillance deficits and astrocytic structural remodeling.

Unlike the clear reduction in microglial density induced by SD, reductions in GFAP expression and GFAP morphological complexity do not necessarily indicate a loss of astrocyte number. As major space‐occupying cells and structural “glue” of the central nervous system, whether SD leads to astrocyte loss versus functional or domain‐level remodeling requires further investigation. The impact of SD on the fine structural organization of astrocytic domains [46] therefore remains an important and unresolved question. Future studies using astrocyte‐specific labeling and three‐dimensional domain‐based analyses will be needed to distinguish true astrocyte loss from reduced GFAP expression or territorial remodeling.

### 4.2 Impact on Synaptic Proteins and Dendritic Spine Morphology: Comparison of SSD and LSD

Both SSD and LSD caused significant reductions in synaptic protein expression and dendritic spine density, with LSD inducing more severe effects. Synaptic proteins such as PSD95 and NMDAR2B were notably reduced in the hippocampus following LSD, suggesting disrupted synaptic integrity. Because PSD95 is a key postsynaptic scaffolding protein and NMDAR2B is involved in glutamatergic synaptic transmission and plasticity, their reduction indicates impairment of both synaptic structure and plasticity‐related signaling. These molecular alterations were accompanied by impairments in hippocampus‐dependent behavioral performance, supporting a functional association between synaptic disruption and cognitive deficits. D30 treatment partially reversed the decrease in synaptic proteins, highlighting its potential to preserve synaptic integrity under conditions of chronic sleep deprivation.

In addition to protein expression, dendritic spine morphology was impaired in both SSD and LSD conditions. Spine density was significantly reduced, with LSD showing more pronounced effects. Given that dendritic spines represent the major postsynaptic sites of excitatory synapses, reduced spine density provides structural evidence for SD‐induced synaptic destabilization. D30 treatment effectively preserved spine density to near control levels, indicating its capacity to support synaptic integrity in the face of chronic sleep loss. However, D30 did not significantly affect spine diameter, suggesting that while D30 can preserve spine quantity, it may not fully promote spine maturation or restore synaptic strength. Thus, D30 appears to exert a stronger protective effect on the maintenance of synaptic structures than on the full restoration of spine maturation or functional synaptic efficacy.

Because Thy1‐EGFP signal reflects promoter‐driven reporter expression, spine analyses were necessarily performed on neurons retaining detectable EGFP signal. This introduces a potential sampling bias toward relatively resilient neuronal subpopulations. However, convergent reductions in PSD95 and NMDAR2B expression provide independent molecular evidence for synaptic impairment under SD and synaptic protection by D30 [47]. Accordingly, dendritic spine data should be interpreted as evidence of structural remodeling in EGFP‐retaining neurons rather than as a complete representation of all hippocampal neurons. Importantly, rather than indicating widespread neuronal or synaptic loss, the reduction in Thy1‐EGFP signal more likely reflects SD‐induced suppression of Thy1 promoter activity, paralleling the observed downregulation of Cx3CR1 and IBA1 transcription in microglia. This interpretation is further supported by the absence of obvious neuronal loss or apoptotic activation in NeuN and cleaved caspase‐3 analyses.

### 4.3 Metabolomic Profiling: Comparison of SSD and LSD

The metabolic consequences of SSD and LSD were investigated using untargeted plasma metabolomics. Hierarchical clustering and volcano plot analyses revealed distinct disruptions in both models. SSD induced transient metabolic changes, primarily involving oxidative stress, lipid metabolism, and amino acid homeostasis. In contrast, LSD caused sustained metabolic dysfunction, marked by downregulation of citric acid and nicotinic acid, indicating impaired TCA cycle activity and mitochondrial dysfunction. Elevated palmitoyl‐L‐carnitine suggested increased fatty acid oxidation as a compensatory response to mitochondrial stress.

Comparison between SSD and LSD highlighted that while SSD triggered short‐term metabolic stress, LSD resulted in more severe and persistent disruptions. Under LSD conditions, metabolic alterations were dominated by mitochondrial dysfunction, lipid metabolic imbalance, oxidative stress, and amino acid dysregulation, consistent with prolonged systemic energy stress. These findings indicate that sleep deprivation duration is a critical determinant of metabolic outcome, with short‐term sleep loss mainly inducing adaptive metabolic fluctuations, whereas prolonged sleep disruption drives sustained metabolic remodeling.

Notably, D30 treatment preserved several key metabolites, including citric acid, L‐kynurenine [48], 1‐methyladenosine, and berberine‐related signals [49] from LSD‐induced reduction, suggesting its potential to rebalance metabolic pathways involved in energy production, neurotransmission, and neuroinflammation. Among these metabolites, citric acid may reflect TCA cycle‐related energy metabolism, whereas L‐kynurenine is linked to tryptophan metabolism and neuroimmune regulation. KEGG pathway analysis confirmed that D30 modulated pathways related to mitochondrial function and amino acid metabolism, indicating its role in restoring metabolic homeostasis and indirectly supporting synaptic function. Because metabolomic profiling was performed in plasma, these changes should be interpreted as systemic metabolic signatures associated with SD and D30 treatment rather than direct evidence of brain‐specific metabolic alterations. Nevertheless, the close relationship among peripheral metabolism, mitochondrial function, neuroinflammation, and cognition supports the possibility that D30 contributes to neuroprotection partly through systemic metabolic stabilization.

In summary, SSD induced short‐term metabolic shifts, whereas LSD led to more enduring systemic metabolic disruption. D30 treatment effectively mitigated these LSD‐associated metabolic disturbances, highlighting its potential to preserve metabolic balance and support neurocognitive function under chronic sleep deprivation. Together with the preservation of glial homeostasis and synaptic integrity, these metabolomic findings suggest that D30 may act through coordinated metabolic, glial, and synaptic regulation.

### 4.4 D30 Treatment Attenuates LSD‐Induced Neuroglial and Synaptic Dysfunction Through Integrated Protective Mechanisms

D30 treatment demonstrated robust neuroprotective effects across multiple biological levels. First, it preserved glial function, as evidenced by the maintenance of microglial density and astrocytic morphology. Specifically, D30 attenuated LSD‐induced reductions in IBA1‐positive signal intensity, microglial branching complexity, GFAP expression, and astrocytic structural organization, suggesting that D30 helps maintain glial homeostasis rather than simply suppressing inflammatory activation. Second, D30 significantly attenuated LSD‐induced reductions in synaptic protein expression and dendritic spine density, supporting its capacity to maintain synaptic integrity under chronic sleep deprivation. This synaptic protection was reflected by the preservation of PSD95 and NMDAR2B expression as well as dendritic spine density. Third, metabolomic analyses revealed that D30 modulated key metabolic pathways—including the TCA cycle, AMPK‐associated signaling, and amino acid metabolism—to partially restore systemic metabolic homeostasis [50]. Together, these findings support an integrated protective model in which D30 counteracts sleep deprivation–associated neurobiological stress through coordinated regulation of glial stability and metabolic balance. Thus, the protective action of D30 is unlikely to depend on a single pathway, but instead appears to involve multi‐level regulation across cellular, synaptic, and metabolic dimensions.

Autophagy is a fundamental process for maintaining cellular homeostasis and is critically involved in the clearance of damaged proteins and organelles; however, its dysregulation is increasingly recognized in age‐related neurodegenerative conditions [51, 52]. In our study, LSD altered the expression of autophagy‐related markers, including LC3 and p62. These changes are interpreted as alterations in autophagy‐associated signaling rather than definitive evidence of enhanced autophagic flux, as lysosomal inhibition–based flux assays were not performed. This distinction is important because changes in LC3 and p62 levels alone cannot fully discriminate between increased autophagosome formation and impaired autophagic degradation. While basal autophagy is neuroprotective, maladaptive upregulation of autophagy‐related responses under chronic stress conditions may contribute to synaptic vulnerability and neuronal dysfunction. Therefore, the LSD‐induced changes in LC3 and p62 are best interpreted as disrupted autophagy‐related homeostatic signaling under prolonged sleep deprivation.

This autophagy‐related dysregulation was accompanied by reduced expression of key synaptic proteins such as PSD95 and NMDAR2B, consistent with cognitive impairments observed in LSD mice. Importantly, D30 treatment preserved the expression of these synaptic markers and normalized LC3 and p62 levels, suggesting suppression of pathological autophagy‐associated responses. The ability of D30 to rebalance autophagy‐related signaling may therefore represent a central mechanism underlying its synaptic protective effects. However, because autophagic flux was not directly measured, we conservatively interpret the effect of D30 as normalization of autophagy‐associated marker expression rather than definitive restoration of autophagic flux. This normalization may reduce chronic stress–related pressure on synaptic components and thereby contribute to the preservation of hippocampal synaptic integrity.

Interestingly, increased autophagic activity and reduced PSD95 expression have also been reported in young mice subjected to SD, often in association with heightened neuroinflammatory responses [53]. However, in our study—consistent with recent findings in middle‐aged models [25]—LSD suppressed microglial activity, suggesting that in aging animals, excessive autophagy and metabolic stress, rather than overt neuroinflammation, may represent a dominant mechanism driving SD‐induced synaptic impairment. This age‐dependent difference may help reconcile divergent findings in the literature, in which sleep deprivation can induce inflammatory microglial activation in young animals but microglial suppression or senescence‐like remodeling in middle‐aged or aged animals. Accordingly, D30 may be particularly beneficial in the aging brain by preserving glial surveillance, stabilizing systemic metabolism, and preventing maladaptive autophagy‐associated synaptic vulnerability.

### 4.5 Limitations and Future Directions

Our findings reveal a global reduction in Thy1‐EGFP expression, suggesting that sleep deprivation (SD) may modulate Thy1 promoter activity. However, the precise molecular mechanisms underlying this effect remain unclear. Although D30 treatment mitigated this reduction, whether this occurs via direct transcriptional regulation, epigenetic modification, or secondary effects of neuronal stress and metabolic dysregulation remains to be determined. Importantly, because Thy1‐EGFP fluorescence is driven by promoter‐dependent reporter expression, reduced EGFP signal should not be directly equated with neuronal loss. Future studies incorporating promoter‐specific reporter assays, conditional genetic models, CRISPR‐based transcriptional regulation, and transcriptomic or epigenomic profiling will be required to elucidate these mechanisms. Such approaches will help determine whether SD suppresses Thy1 transcription directly or indirectly through altered neuronal activity, metabolic stress, or glial‐derived signaling.

A further limitation of the present study is that objective sleep architecture was not directly quantified using EEG/EMG recordings. As a result, specific alterations in sleep stages (e.g., NREM/REM duration or fragmentation) could not be correlated with the observed neuroglial, synaptic, and metabolic changes. Future work integrating polysomnographic monitoring will be essential to disentangle sleep stage–specific effects from the broader physiological consequences of sustained sleep disruption. In particular, EEG/EMG monitoring combined with longitudinal behavioral, histological, and metabolomic assessments would provide a more precise framework for linking sleep architecture disruption to hippocampal vulnerability.

In addition, circulating stress hormones, such as corticosterone, were not measured in the current study. Therefore, potential contributions of hypothalamic–pituitary–adrenal (HPA) axis activation or stress‐related endocrine responses to the observed phenotypes cannot be fully excluded. Although the SD paradigm included daily rest windows and behavioral outcomes were not uniformly indicative of heightened anxiety, future studies incorporating hormonal and peripheral inflammatory assessments will be necessary to more precisely dissociate sleep loss–associated effects from secondary stress responses. Measurements of corticosterone, adrenal weight, peripheral cytokines, and autonomic stress markers would help clarify the relative contribution of sleep loss itself versus nonspecific stress exposure.

Another notable limitation is that D30 was administered concurrently with SD, rather than as a post‐insult intervention. While our findings demonstrate a preventive neuroprotective effect of D30 during ongoing sleep deprivation, its therapeutic potential in reversing established SD‐induced deficits remains unknown. Future studies employing post‐SD intervention paradigms will be critical to determine whether D30 primarily prevents or can also rescue sleep deprivation–induced pathology. Such studies should compare preventive, delayed‐treatment, and withdrawal designs to define the therapeutic window, durability of protection, and reversibility of glial, synaptic, and metabolic abnormalities.

Finally, this study exclusively utilized male mice, which limits generalizability. Male animals were selected to minimize variability associated with estrous cycle–dependent hormonal fluctuations, as estrogens are known to modulate neuroinflammatory responses, synaptic plasticity, and metabolic regulation. Moreover, sex‐related differences in metabolic vulnerability and synaptic remodeling have been reported, with male mice often exhibiting greater susceptibility to metabolic stress. Nevertheless, we acknowledge that the exclusion of females restricts translational relevance. Future investigations should include both sexes and adopt multifactorial designs to comprehensively evaluate sex‐specific responses and sex–treatment interactions. Including female mice across defined estrous stages, or using appropriately powered mixed‐sex cohorts, will be important for determining whether D30 confers comparable protection across sexes.

In addition to these limitations, the present study mainly focused on histological, biochemical, behavioral, and plasma metabolomic endpoints. Direct causal links among glial suppression, synaptic impairment, autophagy‐related signaling, and systemic metabolic remodeling remain to be established. Future mechanistic studies using cell type–specific manipulations, region‐specific metabolic profiling, and in vivo functional assays will be needed to determine how these processes interact during chronic sleep deprivation and D30 treatment.

## 5 Conclusion

Our findings demonstrate that both SSD and LSD disrupt glial homeostasis, alter synaptic protein expression, and perturb systemic metabolism in middle‐aged mice, with LSD producing more severe and persistent effects. Compared with SSD, LSD induced more pronounced microglial and astrocytic dysfunction, synaptic protein loss, dendritic spine deterioration, and mitochondrial dysregulation. The small molecule D30 effectively counteracted LSD‐induced deficits by preserving glial homeostatic features, restoring synaptic protein expression, normalizing autophagy‐associated marker expression, and rebalancing metabolic pathways. These multimodal actions significantly ameliorated LSD‐induced cognitive decline. This study provides mechanistic insight into how sleep deprivation duration shapes neuroglial, synaptic, and metabolic vulnerability and highlights D30 as a promising candidate for the prevention or management of chronic sleep deprivation–related cognitive impairment.

## Author Contributions

**Xueyan Liu** and **Zu‐Cheng Ye** designed the project; **Xueyan Liu**, **Ping Chen**, and **Zu‐Cheng Ye** interpreted the data and wrote the manuscript; **Huiling Lin** and **Xili Chen** performed WB experiment; **Jian Zhong** performed IF experiment; **Xueyan Liu**, **Hui Zhang**, and **Jian Zhong** performed data analysis; **Huiling Lin** and **Jian Zhong** performed mouse behavioral test; **Zonglin Wang**, **Meng Shi**, and **Hang Shi** participated in discussion of the project and implementation; **Zu‐Cheng Ye** edited and finalized the manuscript. All the authors read and approved the final manuscript.

## Funding

This research was supported by grants from the Fujian Provincial Natural Science Foundation, China (Grant# 2021J02032 to Y.Z.); the Fujian Provincial Natural Science Foundation, China (Grant# 2024J01509 to L.X.); the Open Project of Fujian Key Laboratory of Molecular Neurology, China (Grant# 2023‐SJKF‐003 to L.X.); the Open Project of Fujian Key Laboratory of Brain Aging and Neurodegenerative Diseases, China (Grant# 2023BAND02 to L.X.); the Open Project of State Key Laboratory of Supramolecular Structure and Materials, China (Grant# sklssm2024015 to L.X.).

## Ethics Statement

All animal studies were performed with the approval of the Institutional Animal Care and Use Committee (IACUC) of Fujian Medical University (IACUC FJMU 2024‐0274).

## Conflicts of Interest

The authors declare no conflicts of interest.

## Supporting information


**Figure S1:** Effects of SSD and D30 treatment on Thy1‐EGFP expression and microglial marker expression in Thy1‐EGFP and C57BL/6 mice. (A) Representative images of hippocampal Thy1‐EGFP fluorescence (green), IBA1 immunostaining (red), DAPI staining (blue), and merged signals in Ctrl, Ctrl+D30, SSD, and SSD+D30 Thy1‐EGFP mice. Scale bars, 500 μm. (B) Schematic diagram of the 6‐day SSD protocol and D30 administration. (C) Quantification of Thy1 mRNA expression in the hippocampus of C57BL/6 mice under control and SSD conditions. (D, E) Quantification of Iba1 (D) and Cx3cr1 (E) mRNA expression in the hippocampus of C57BL/6 mice under Ctrl, Ctrl+D30, SSD, and SSD+D30 conditions. Data are presented as mean ± SEM, with each dot representing one mouse (*n* = 6 mice per group in C and *n* = 5 mice per group in D and E). Statistical significance in C was assessed using an unpaired two‐tailed Student's *t*‐test. For D and E, statistical significance was assessed using ordinary two‐way ANOVA with experimental condition (Ctrl versus SSD) and treatment (vehicle versus D30) as factors, followed by Šídák's multiple‐comparisons test. For D, the main effects of experimental condition [*F*(1, 16) = 24.32, *p* = 0.0002] and treatment [*F*(1, 16) = 4.716, *p* = 0.0453], as well as their interaction [*F*(1, 16) = 10.70, *p* = 0.0048], were significant. For E, the main effects of experimental condition [*F*(1, 16) = 5.323, *p* = 0.0348] and treatment [*F*(1, 16) = 8.632, *p* = 0.0096] were significant, whereas their interaction was not significant [*F*(1, 16) = 0.5109, *p* = 0.4851]. ns, not significant; *p < 0.05, **p < 0.01, ***p < 0.001, and ****p < 0.0001.
**Figure S2:** Quantification of IBA1‐positive microglial profile‐associated signals in CA1 region of Cx3CR1‐EGFP mice. (A) Experimental timeline for SSD intervention. (B) Representative immunofluorescence images of IBA1‐positive microglia (red) in the CA1 region from control and SSD‐treated mice. (C) Quantification of IBA1‐positive microglial density in CA1. (D, F) High‐magnification images of microglia from control (D) and SSD‐treated (F) mice. (E, G) Sholl analysis of microglial morphology showing (E) number of branches and (G) maximum branch length. Data are shown as mean ± SEM (*n* = 5 animals per group). Statistical significance was assessed using unpaired two‐tailed *t*‐tests. ns, not significant; *p < 0.05, **p < 0.01, ***p < 0.001, ****p < 0.0001.
**Figure S3:** Three‐dimensional reconstructed images of microglial morphology in the DG and CA1 regions of the hippocampus from Ctrl and SSD Cx3cr1‐GFP transgenic mice. Representative 3D reconstructed images of Cx3cr1‐GFP‐positive microglia in the DG and CA1 regions under Ctrl and SSD conditions. Images were acquired and displayed under identical microscopic settings. Scale bar: 10 μm.
**Figure S4:** Plasma metabolomic profiling of 12‐month‐old Thy1‐EGFP mice following SSD. (A) Hierarchical clustering heatmap of significantly altered metabolites in the Ctrl and SSD groups, showing distinct metabolic profiles. (B) Volcano plot illustrating significantly upregulated (red) and downregulated (blue) metabolites in SSD versus controls. (C) KEGG pathway enrichment analysis revealing major metabolic pathways impacted by SSD, including oxidative phosphorylation, TCA cycle, and amino acid metabolism. (D) KEGG network diagram highlighting key dysregulated metabolic hubs and pathway interactions in SSD‐treated mice.
**Figure S5:** General health‐, activity‐, and mobility‐related control parameters in LSD‐exposed 12‐month‐old Thy1‐EGFP mice. (A) Body weight changes during the 28‐day LSD paradigm. (B) Daily food consumption during the LSD period. (C) Total distance traveled in the open field test. (D) Total exploration time in the novel object recognition test. (E) Swimming speed in the Morris water maze test. (F) Total swimming distance in the Morris water maze test. Data are presented as mean ± SEM, with each dot representing one mouse in C–F (*n* = 10 mice per group). Statistical significance in C–F was assessed using ordinary two‐way ANOVA with experimental condition (Ctrl versus LSD) and treatment (vehicle versus D30) as factors, followed by Šídák's multiple‐comparisons test. For C, neither the main effect of experimental condition [*F*(1, 36) = 9.303 × 10^−6^, *p* = 0.9976], the main effect of treatment [*F*(1, 36) = 1.707, *p* = 0.1997], nor their interaction [*F*(1, 36) = 1.437, *p* = 0.2384] was significant. For D, neither the main effect of experimental condition [*F*(1, 36) = 3.149, *p* = 0.0844], the main effect of treatment [*F*(1, 36) = 0.9407, *p* = 0.3386], nor their interaction [*F*(1, 36) = 1.704, *p* = 0.2000] was significant. For E, the main effect of D30 treatment was significant [*F*(1, 36) = 13.36, *p* = 0.0008], whereas the main effect of experimental condition [*F*(1, 36) = 3.889, *p* = 0.0563] and their interaction [*F*(1, 36) = 1.198, *p* = 0.2810] were not significant. For F, the main effect of D30 treatment was significant [*F*(1, 36) = 18.97, *p* = 0.0001], whereas the main effect of experimental condition [*F*(1, 36) = 1.560, *p* = 0.2197] and their interaction [*F*(1, 36) = 0.9378, *p* = 0.3393] were not significant. ns, not significant; *p < 0.05, **p < 0.01, ***p < 0.001, and ****p < 0.0001.
**Figure S6:** LSD reduces Thy1‐EGFP expression without inducing overt neuronal loss or caspase‐3‐dependent neuronal apoptosis in Thy1‐EGFP mice. (A) Representative whole‐brain fluorescence images showing Thy1‐EGFP expression in control (Ctrl), Ctrl+D30, long‐term sleep‐deprived (LSD), and LSD+D30 Thy1‐EGFP mice. LSD markedly reduced Thy1‐EGFP fluorescence intensity throughout the brain, whereas D30 treatment attenuated this reduction and restored EGFP signal intensity toward control levels. (B) Quantification of relative Thy1‐EGFP fluorescence intensity from panel A. (C) Representative immunofluorescence images of NeuN‐positive neurons, cleaved caspase‐3, DAPI, and merged signals in the dentate gyrus (DG). (D) Quantification of NeuN‐positive neuronal density in the DG. (E) Quantification of cleaved caspase‐3^+^NeuN^+^ cells as a percentage of total NeuN^+^ cells in the DG. No significant changes in NeuN‐positive neuronal density or cleaved caspase‐3^+^NeuN^+^ cells were observed following LSD, indicating that the LSD‐induced reduction in Thy1‐EGFP fluorescence was unlikely to be attributable to overt neuronal loss or caspase‐3‐dependent neuronal apoptosis. Scale bars, 500 μm in A and 25 μm in C. Data are presented as mean ± SEM, with each dot representing one mouse (*n* = 5 mice per group). Statistical significance was assessed using ordinary two‐way ANOVA with experimental condition (Ctrl versus LSD) and treatment (vehicle versus D30) as factors, followed by Šídák's multiple‐comparisons test. For B, the main effects of experimental condition [*F*(1, 16) = 63.79, *p* < 0.0001] and treatment [*F*(1, 16) = 115.1, *p* < 0.0001], as well as their interaction [*F*(1, 16) = 81.33, *p* < 0.0001], were significant. For D, neither the main effect of experimental condition [*F*(1, 16) = 3.537, *p* = 0.0783], the main effect of treatment [*F*(1, 16) = 1.951, *p* = 0.1816], nor their interaction [*F*(1, 16) = 4.316, *p* = 0.0542] was significant. For E, neither the main effect of experimental condition [*F*(1, 16) = 0.3757, *p* = 0.5485], the main effect of treatment [*F*(1, 16) = 2.381, *p* = 0.1424], nor their interaction [*F*(1, 16) = 1.255, *p* = 0.2792] was significant. ns, not significant; *p < 0.05, **p < 0.01, ***p < 0.001, and ****p < 0.0001.
**Figure S7:** Effects of D30 treatment on synaptic protein expression and autophagy‐related markers in the hippocampus following LSD. (A–C) Representative immunofluorescence images of PSD95‐positive signals in the DG and CA1 regions (A), with corresponding quantification in the DG (B) and CA1 (C). (D) Representative western blots of PSD95, NMDAR2A, NMDAR2B, synaptophysin, CaMKII, LC3‐I/II, p62, and ACTIN in hippocampal tissue. (E–K) Densitometric analysis of western blot bands showing relative protein levels of PSD95 (E), NMDAR2A (F), NMDAR2B (G), synaptophysin (H), CaMKII (I), the LC3‐II/LC3‐I ratio (J), and p62 (K). Protein levels were normalized to ACTIN, except for the LC3‐II/LC3‐I ratio. Scale bars, 3 μm in A. Data are presented as mean ± SEM, with each dot representing one mouse; *n* = 5 mice per group for immunofluorescence quantification and *n* = 3 mice per group for western blot analysis. Statistical significance was assessed using ordinary two‐way ANOVA with experimental condition (Ctrl vs. LSD) and treatment (vehicle versus D30) as factors, followed by Šídák's multiple‐comparisons test. For B, the main effects of experimental condition [*F*(1, 16) = 51.53, *p* < 0.0001] and treatment [*F*(1, 16) = 29.21, *p* < 0.0001], as well as their interaction [*F*(1, 16) = 9.948, *p* = 0.0061], were significant. For C, the main effect of experimental condition [*F*(1, 16) = 14.30, *p* = 0.0016] and the condition × treatment interaction [*F*(1, 16) = 9.362, *p* = 0.0075] were significant, whereas the main effect of treatment was not significant [*F*(1, 16) = 3.814, *p* = 0.0685]. For E, the condition × treatment interaction was significant [*F*(1, 8) = 13.50, *p* = 0.0063], whereas the main effects of experimental condition [*F*(1, 8) = 1.381, *p* = 0.2737] and treatment [*F*(1, 8) = 1.192, *p* = 0.3067] were not significant. For F, the main effect of experimental condition was significant [*F*(1, 8) = 31.95, *p* = 0.0005], whereas the main effect of treatment [*F*(1, 8) = 1.301, *p* = 0.2870] and the interaction [*F*(1, 8) = 1.058, *p* = 0.3338] were not significant. For G, the main effect of experimental condition [*F*(1, 8) = 35.24, *p* = 0.0003] and the condition × treatment interaction [*F*(1, 8) = 11.35, *p* = 0.0098] were significant, whereas the main effect of treatment was not significant [*F*(1, 8) = 4.544, *p* = 0.0656]. For H, the main effects of experimental condition [*F*(1, 8) = 11.20, *p* = 0.0101] and treatment [*F*(1, 8) = 13.77, *p* = 0.0060] were significant, whereas their interaction was not significant [*F*(1, 8) = 3.118, *p* = 0.1154]. For I, the main effects of experimental condition [*F*(1, 8) = 105.6, *p* < 0.0001] and treatment [*F*(1, 8) = 177.4, *p* < 0.0001], as well as their interaction [*F*(1, 8) = 19.10, *p* = 0.0024], were significant. For J, the main effects of experimental condition [*F*(1, 8) = 57.90, *p* < 0.0001] and treatment [*F*(1, 8) = 30.05, *p* = 0.0006], as well as their interaction [*F*(1, 8) = 26.62, *p* = 0.0009], were significant. For K, the main effect of experimental condition [*F*(1, 8) = 6.324, *p* = 0.0361] and the condition × treatment interaction [*F*(1, 8) = 20.48, *p* = 0.0019] were significant, whereas the main effect of treatment was not significant [*F*(1, 8) = 0.2350, *p* = 0.6409]. ns, not significant; *p < 0.05, **p < 0.01, ***p < 0.001, and ****p < 0.0001.
**Figure S8:** KEGG pathway enrichment analysis of mice following LSD and D30 treatment. (A) KEGG pathway analysis comparing LSD versus Ctrl groups, identifying key metabolic pathways significantly affected by LSD. (B) KEGG pathway analysis comparing LSD+D30 versus LSD groups, illustrating metabolic pathway modulation by D30 treatment.

## Data Availability

Data will be made available on request.
