# Supplementary material for: Sleep Deprivation Induces Glial Dysfunction, Synaptic Loss, Metabolic Imbalance, and Cognitive Impairment in 12‐Month‐Old Mice: Protective Effects of D30
Source: CNS Neurosci Ther. 2026 Sep 10;32(9):e71134. doi: 10.1002/cns.71134 (PMC13560928; doi:10.1002/cns.71134)
Supplement: Supplementary file 1 [file CNS-32-e71134-s001.docx]

**Sleep deprivation induces glial dysfunction, synaptic loss, metabolic imbalance, and cognitive impairment in 12-month-old mice: Protective effects of D30**

Xueyan Liu^a, b, #^, Huiling Lin^b, #^, Jian Zhong^b, #^, Xili Chen^b^, Zonglin Wang^b^, Meng Shi^b^, Hang Shi^b^, Hui Zhang^c^, Ping Chen^b, d, **^, Zu-Cheng Ye^b,*^

^a^ Fujian Medical University School of Pharmacy, Fuzhou, 350112, Fujian Province, China.

^b^Fujian Medical University School of Basic Medical Sciences, Fuzhou, 350112, Fujian Province, China.

^c^Fujian Medical University, Fuzhou 350112, Fujian Province, China

^d^First Affiliated Hospital of Fujian Medical University Department of Anesthesiology, Fuzhou, 350005, Fujian Province, China.

^**^ Corresponding author. E-mail address: cp1118@fjmu.edu.cn

^*^ Corresponding author. E-mail address: [zcye@fjmu.edu.cn](mailto:xueyanliu@fjmu.edu.cn)

^#^ These authors contributed equally to this work.

**Figure S1. Effects of SSD and D30 treatment on Thy1-EGFP expression and microglial marker expression in Thy1-EGFP and C57BL/6 mice.** (A) Representative images of hippocampal Thy1-EGFP fluorescence (green), IBA1 immunostaining (red), DAPI staining (blue), and merged signals in Ctrl, Ctrl+D30, SSD, and SSD+D30 Thy1-EGFP mice. Scale bars, 500 μm. (B) Schematic diagram of the 6-day SSD protocol and D30 administration. (C) Quantification of Thy1 mRNA expression in the hippocampus of C57BL/6 mice under control and SSD conditions. (D, E) Quantification of Iba1 (D) and Cx3cr1 (E) mRNA expression in the hippocampus of C57BL/6 mice under Ctrl, Ctrl+D30, SSD, and SSD+D30 conditions. Data are presented as mean ± SEM, with each dot representing one mouse (n = 6 mice per group in C and n = 5 mice per group in D and E). Statistical significance in C was assessed using an unpaired two-tailed Student’s t-test. For D and E, statistical significance was assessed using ordinary two-way ANOVA with experimental condition (Ctrl versus SSD) and treatment (vehicle versus D30) as factors, followed by Šídák’s multiple-comparisons test. For D, the main effects of experimental condition [F(1, 16) = 24.32, P = 0.0002] and treatment [F(1, 16) = 4.716, P = 0.0453], as well as their interaction [F(1, 16) = 10.70, P = 0.0048], were significant. For E, the main effects of experimental condition [F(1, 16) = 5.323, P = 0.0348] and treatment [F(1, 16) = 8.632, P = 0.0096] were significant, whereas their interaction was not significant [F(1, 16) = 0.5109, P = 0.4851]. ns, not significant; ^*^P < 0.05, ^**^P < 0.01, ^***^P < 0.001, and ^****^P < 0.0001.

**Figure S2. Quantification of IBA1-positive microglial profile-associated signals in CA1 region of Cx3CR1-EGFP mice.** (A) Experimental timeline for SSD intervention. (B) Representative immunofluorescence images of IBA1-positive microglia (red) in the CA1 region from control and SSD-treated mice. (C) Quantification of IBA1-positive microglial density in CA1. (D, F) High-magnification images of microglia from control (D) and SSD-treated (F) mice. (E, G) Sholl analysis of microglial morphology showing (E) number of branches and (G) maximum branch length. Data are shown as mean ± SEM (n = 5 animals per group). Statistical significance was assessed using unpaired two-tailed t-tests. ns, not significant; ^*^*P* < 0.05, ^**^ *P* < 0.01, ^***^ *P* < 0.001, ^****^ *P* < 0.0001.

**Figure S3. Three-dimensional reconstructed images of microglial morphology in the DG and CA1 regions of the hippocampus from Ctrl and SSD Cx3cr1-GFP transgenic mice.** Representative 3D reconstructed images of Cx3cr1-GFP-positive microglia in the DG and CA1 regions under Ctrl and SSD conditions. Images were acquired and displayed under identical microscopic settings. Scale bar: 10 μm.

**Figure S4.** **Plasma metabolomic profiling of 12-month-old Thy1-EGFP mice following SSD**. (A) Hierarchical clustering heatmap of significantly altered metabolites in the Ctrl and SSD groups, showing distinct metabolic profiles. (B) Volcano plot illustrating significantly upregulated (red) and downregulated (blue) metabolites in SSD versus controls. (C) KEGG pathway enrichment analysis revealing major metabolic pathways impacted by SSD, including oxidative phosphorylation, TCA cycle, and amino acid metabolism. (D) KEGG network diagram highlighting key dysregulated metabolic hubs and pathway interactions in SSD-treated mice.

**Figure S5. General health-, activity-, and mobility-related control parameters in LSD-exposed 12-month-old Thy1-EGFP mice.** (A) Body weight changes during the 28-day LSD paradigm. (B) Daily food consumption during the LSD period. (C) Total distance traveled in the open field test. (D) Total exploration time in the novel object recognition test. (E) Swimming speed in the Morris water maze test. (F) Total swimming distance in the Morris water maze test. Data are presented as mean ± SEM, with each dot representing one mouse in C–F (n = 10 mice per group). Statistical significance in C–F was assessed using ordinary two-way ANOVA with experimental condition (Ctrl versus LSD) and treatment (vehicle versus D30) as factors, followed by Šídák’s multiple-comparisons test. For C, neither the main effect of experimental condition [F(1, 36) = 9.303 × 10⁻⁶, P = 0.9976], the main effect of treatment [F(1, 36) = 1.707, P = 0.1997], nor their interaction [F(1, 36) = 1.437, P = 0.2384] was significant. For D, neither the main effect of experimental condition [F(1, 36) = 3.149, P = 0.0844], the main effect of treatment [F(1, 36) = 0.9407, P = 0.3386], nor their interaction [F(1, 36) = 1.704, P = 0.2000] was significant. For E, the main effect of D30 treatment was significant [F(1, 36) = 13.36, P = 0.0008], whereas the main effect of experimental condition [F(1, 36) = 3.889, P = 0.0563] and their interaction [F(1, 36) = 1.198, P = 0.2810] were not significant. For F, the main effect of D30 treatment was significant [F(1, 36) = 18.97, P = 0.0001], whereas the main effect of experimental condition [F(1, 36) = 1.560, P = 0.2197] and their interaction [F(1, 36) = 0.9378, P = 0.3393] were not significant. ns, not significant; ^*^P < 0.05, ^**^P < 0.01, ^***^P < 0.001, and ^****^P < 0.0001.

**Figure S6. LSD reduces Thy1-EGFP expression without inducing overt neuronal loss or caspase-3-dependent neuronal apoptosis in Thy1-EGFP mice.** (A) Representative whole-brain fluorescence images showing Thy1-EGFP expression in control (Ctrl), Ctrl+D30, long-term sleep-deprived (LSD), and LSD+D30 Thy1-EGFP mice. LSD markedly reduced Thy1-EGFP fluorescence intensity throughout the brain, whereas D30 treatment attenuated this reduction and restored EGFP signal intensity toward control levels. (B) Quantification of relative Thy1-EGFP fluorescence intensity from panel A. (C) Representative immunofluorescence images of NeuN-positive neurons, cleaved caspase-3, DAPI, and merged signals in the dentate gyrus (DG). (D) Quantification of NeuN-positive neuronal density in the DG. (E) Quantification of cleaved caspase-3⁺NeuN⁺ cells as a percentage of total NeuN⁺ cells in the DG. No significant changes in NeuN-positive neuronal density or cleaved caspase-3⁺NeuN⁺ cells were observed following LSD, indicating that **the** LSD-induced **reduction** in Thy1-EGFP fluorescence **was** unlikely to be attributable to overt neuronal loss or caspase-3-dependent neuronal apoptosis. **Scale bars, 500 μm in A and 25 μm in C.** Data are presented as mean ± SEM, **with each dot representing one mouse** (n = 5 **mice** per group). Statistical significance was **assessed using ordinary** two-way ANOVA **with experimental condition (Ctrl versus LSD) and treatment (vehicle versus D30) as factors**, followed by Šídák’s multiple-comparisons test. **For B, the main effects of experimental condition [**F**(1, 16) = 63.79,** P **< 0.0001] and treatment [**F**(1, 16) = 115.1,** P **< 0.0001], as well as their interaction [**F**(1, 16) = 81.33,** P **< 0.0001], were significant. For D, neither the main effect of experimental condition [**F**(1, 16) = 3.537,** P **= 0.0783], the main effect of treatment [**F**(1, 16) = 1.951,** P **= 0.1816], nor their interaction [**F**(1, 16) = 4.316,** P **= 0.0542] was significant. For E, neither the main effect of experimental condition [**F**(1, 16) = 0.3757,** P **= 0.5485], the main effect of treatment [**F**(1, 16) = 2.381,** P **= 0.1424], nor their interaction [**F**(1, 16) = 1.255,** P **= 0.2792] was significant.** ns, not significant; ^*^P < 0.05, ^**^P < 0.01, ^***^P < 0.001, and ^****^P < 0.0001.

**Figure S7. Effects of D30 treatment on synaptic protein expression and autophagy-related markers in the hippocampus following LSD.** (A–C) Representative immunofluorescence images of **PSD95-positive signals** in the DG and CA1 regions (A), with corresponding quantification in the DG (B) and CA1 (C). (D) **Representative western blots of** PSD95, NMDAR2A, NMDAR2B, synaptophysin, CaMKII, LC3-I/II, p62, **and ACTIN** in hippocampal tissue. (E–K) Densitometric analysis of western blot bands showing relative protein levels of PSD95 (E), NMDAR2A (F), NMDAR2B (G), synaptophysin (H), CaMKII (I), **the** LC3-II/LC3-I ratio (J), and p62 (K). Protein levels were normalized to ACTIN, except for the LC3-II/LC3-I ratio. **Scale bars, 3 μm in A.** Data are presented as mean ± SEM, **with each dot representing one mouse**; n = 5 **mice** per group for immunofluorescence quantification and n = 3 **mice** per group for western blot analysis. Statistical significance was **assessed using ordinary** two-way ANOVA **with experimental condition (Ctrl versus LSD) and treatment (vehicle versus D30) as factors**, followed by Šídák’s multiple-comparisons test. **For B, the main effects of experimental condition [**F**(1, 16) = 51.53,** P **< 0.0001] and treatment [**F**(1, 16) = 29.21,** P **< 0.0001], as well as their interaction [**F**(1, 16) = 9.948,** P **= 0.0061], were significant. For C, the main effect of experimental condition [**F**(1, 16) = 14.30,** P **= 0.0016] and the condition × treatment interaction [**F**(1, 16) = 9.362,** P **= 0.0075] were significant, whereas the main effect of treatment was not significant [**F**(1, 16) = 3.814,** P **= 0.0685]. For E, the condition × treatment interaction was significant [**F**(1, 8) = 13.50,** P **= 0.0063], whereas the main effects of experimental condition [**F**(1, 8) = 1.381,** P **= 0.2737] and treatment [**F**(1, 8) = 1.192,** P **= 0.3067] were not significant. For F, the main effect of experimental condition was significant [**F**(1, 8) = 31.95,** P **= 0.0005], whereas the main effect of treatment [**F**(1, 8) = 1.301,** P **= 0.2870] and the interaction [**F**(1, 8) = 1.058,** P **= 0.3338] were not significant. For G, the main effect of experimental condition [**F**(1, 8) = 35.24,** P **= 0.0003] and the condition × treatment interaction [**F**(1, 8) = 11.35,** P **= 0.0098] were significant, whereas the main effect of treatment was not significant [**F**(1, 8) = 4.544,** P **= 0.0656]. For H, the main effects of experimental condition [**F**(1, 8) = 11.20,** P **= 0.0101] and treatment [**F**(1, 8) = 13.77,** P **= 0.0060] were significant, whereas their interaction was not significant [**F**(1, 8) = 3.118,** P **= 0.1154]. For I, the main effects of experimental condition [**F**(1, 8) = 105.6,** P **< 0.0001] and treatment [**F**(1, 8) = 177.4,** P **< 0.0001], as well as their interaction [**F**(1, 8) = 19.10,** P **= 0.0024], were significant. For J, the main effects of experimental condition [**F**(1, 8) = 57.90,** P **< 0.0001] and treatment [**F**(1, 8) = 30.05,** P **= 0.0006], as well as their interaction [**F**(1, 8) = 26.62,** P **= 0.0009], were significant. For K, the main effect of experimental condition [**F**(1, 8) = 6.324,** P **= 0.0361] and the condition × treatment interaction [**F**(1, 8) = 20.48,** P **= 0.0019] were significant, whereas the main effect of treatment was not significant [**F**(1, 8) = 0.2350,** P **= 0.6409].** ns, not significant; ^*^P < 0.05, ^**^P < 0.01, ^***^P < 0.001, **and** ^****^P < 0.0001.

**Figure S8. KEGG pathway enrichment analysis of mice following LSD and D30 treatment.** A. KEGG pathway analysis comparing LSD vs. Ctrl groups, identifying key metabolic pathways significantly affected by LSD. B. KEGG pathway analysis comparing LSD + D30 vs. LSD groups, illustrating metabolic pathway modulation by D30 treatment.
